# Synthesis of 86 species of 1,5-diaryl-3-oxo-1,4-pentadienes analogs of curcumin can yield a good lead *in vivo*

**DOI:** 10.1186/1471-2210-11-4

**Published:** 2011-05-28

**Authors:** Chieko Kudo, Hiroyuki Yamakoshi, Atsuko Sato, Hiroshi Nanjo, Hisatsugu Ohori, Chikashi Ishioka, Yoshiharu Iwabuchi, Hiroyuki Shibata

**Affiliations:** 1 Dept. Clinical Oncology, Institute of Development, Aging, and Cancer, Tohoku University, Seiryo-cho 4-1, Aoba-ku, Sendai, Japan; 2 Dept. Clinical Oncology, University Hospital, Tohoku University, Seiryo-cho 1-1, Aoba-ku, Sendai, Japan; 3 Dept. Organic Chemistry, Graduate School of Pharmaceutical Science, Tohoku University, Aoba 6-3, Aramaki, Aoba-ku, Sendai, Japan; 4 Division of Clinical Pathology, Faculty of Medicine, Akita University, Hondo1-1-1, Akita, Japan; 5 Dept. Clinical Oncology, Faculty of Medicine, Akita University, Hondo1-1-1, Akita, Japan

## Abstract

**Background:**

Curcumin is known to possess many anti-tumor properties such as inhibition of tumor growth and induction of apotosis. However, limited bioavailability of curcumin prevents its clinical application. A synthesized curcumin analog, 1,5-diaryl-3-oxo-1,4-pentadiene such as GO-Y030, has the improved anti-tumor potential *in vitro *as well as in mouse model of colorectal carcinogenesis.

**Results:**

These compounds were divided into two groups; one is the higher anti-proliferative group, in which 79.7% of 1,5-diaryl-3-oxo-1,4-pentadienes were clustered. One of the 1,5-diaryl-3-oxo-1,4-pentadiene analogs, GO-Y078 has the most enhanced growth inhibition, and its solubility was improved, compared with curcumin. GO-Y078 inhibits NF-κB transactivation, as well as expression of TP53 and DR5 more effectively than curcumin. In a mouse model, GO-Y078 presented 1.4 fold more survival elongation that was not achieved by curcumin and GO-Y030.

**Conclusions:**

The 1,5-diaryl-3-oxo-1,4-pentadiene analogs can yield good lead compounds for cancer chemotherapy, to overcome low bioavailability of curcumin.

## Background

Naturally derived products are the most valuable source for drugs and their lead compounds. Approximately 74% of anticancer drugs are either natural products or their derivatives [1]. Curcumin, 1,7-bis(4-hydroxy-3-methoxyphenyl)-1,6-heptadiene-3,5-dione, is a dietary constituent of turmeric. It is well known for its ability to suppress tumor growth [2]. The mechanisms of its tumor suppression have been examined at the molecular level. Curcumin is known to interfere with the transactivation of nuclear factor-κB (NF-κB) [3], activator protein 1 [4], and β-catenin [5] thus resulting in the negative regulation of various oncogenes such as *c-Myc, cyclin D1, Bcl-2*, and *Bcl-XL*. Curcumin arrests the cell cycle at G_0_/G_1 _and/or G_2_/M through the upregulation of the cyclin-dependent kinase inhibitors p21 and p27, and the downregulation of Cdc2 and cyclin B1 [6]. Curcumin blocks signaling of growth factors, including human epidermal growth factor receptor-2 [7], platelet-derived growth factor [8] and fibroblast growth factor [9]. It also blocks *Wnt *signalling [10]. Curcumin also has anti-invasive, antimetastastic, and antiangiogenic properties [11,12]. Therefore, curcumin is labeled as a multi-targeted drug. However, the systemic availability of curcumin remains negligible [13,14]. Hydrophobicity, low absorption, and rapid metabolism have been considered as reasons for curcumin's low bioavailability [2]. To address this issue, we synthesized a series of curcumin analogs and screened them. An analog named GO-Y030 was synthesized that possessed 30- to 50-fold enhanced antiproliferative potential against various types of cancers *in vitro *[15]. The molecular mechanisms of GO-Y030 closely resembled those of curcumin. Oral administration of GO-Y030 prevented the adenoma formation in the familial adenomatous polyposis (FAP) mouse model without any apparent toxicity [16]. It was also demonstrated that the number and the size of β-catenin-positive adenomas were reduced. In this case, bioavailability of GO YO30 was not an issue because it can be directly delivered to the gastrointestinal tract. However, GO-Y030 was hydrophobic and ineffective to the treatment of peritoneal carcinomatosis (PC) of gastric carcinoma in a mouse model. These observations led us to additionally synthesize hyperactive curcumin analogs in order to optimize their pharmacological potential.

## Results

### Screening of the hyperactive growth inhibitors among the newly synthesized curcumin analogs

**Figure 1:**
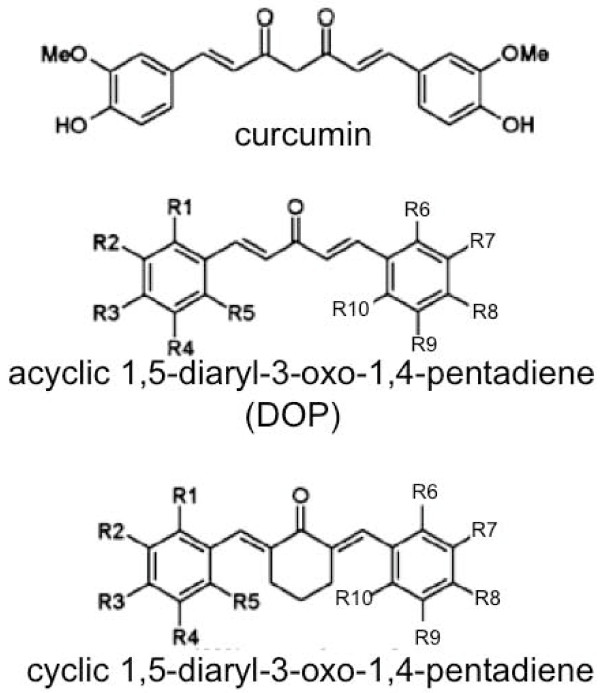
**Chemical structures of curcuminoids**. Two types of basic structure of 1,5-diaryl-3-oxo-1,4-pentadienes are presented.

**Figure 2:**
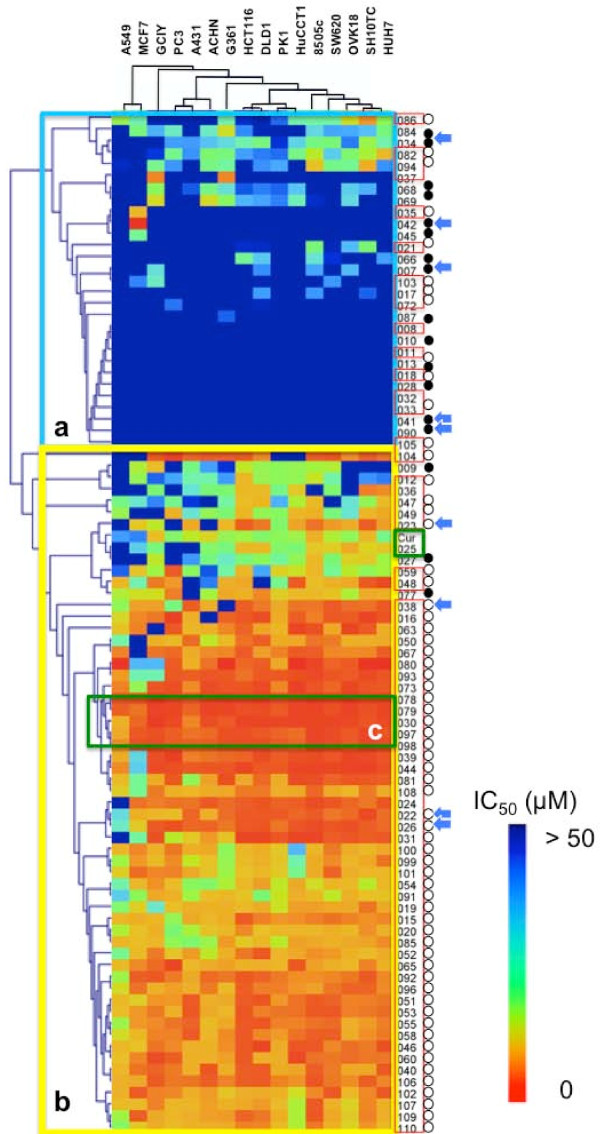
**Cluster analysis of growth inhibition of 88 curcuminoids**. The IC50 values are graded by coloring from red (0 μM) to cyan (50 μM). Eighty eight analogs are lined in perpendicular, and 16 cancer cell lines are lined in horizontal. Group a is composed of the analogs with the high IC_50 _values, and Group b composed of analogs with the low IC_50 _values, and Group c includes the analogs with the lowest IC_50 _values. The analogs enclosed with red rectangle are DOPs, and the analogs attached with closed circle are non-DOPs. Acyclic DOPs are indicated by open circle. Blue arrows indicate the analogs of which predictive values of solubility are better than curcumin.

Eighty eight curcumin analogs, including 69 species of DOPs, curcumin and 1,7-diaryl-1,6-heptadiene-3,5-dione, and 17 species of non-DOP analogs were synthesized (Figure 1). The growth-suppressive potential of these compounds was then examined on cell panels composed of 16 types of cancers. These included gastric cancers (GCIY and SH10TC), colon cancers (HCT116, DLD1 and SW680), lung cancer (A549), pancreatic cancer (PK1), kidney cancer (ACHN), liver cancer (HUH7), ovarian cancer (OVK18), breast cancer (MCF7), skin cancer (A431), bile duct cancer (HuCCT1), thyroid cancer (8505c), melanoma (G361), and prostate cancer (PC3). The IC50 value of each analog was calculated against the individual cell line. These values were analyzed using cluster analysis (Figure 2). Resulting from the pattern of inhibition, 88 curcuminoids were divided into 2 clusters; one is the high IC_50 _group (Group a) composed of 29 analogs, and the other is the low IC_50 _group (Group b) composed of 59 analogs. In detail of Group a, 51.7% (15/29) of those are DOPs, whereas 91.5% (54/59) are DOPs in Group b. From the view point of DOPs, 55 out of 69 (79.7%) belong to Group b. In this study, 64 species out of 69 analogs are classified to acyclic DOPs, and 81.3% species (52/64) of acyclic DOPs belong to Group b. On the other hand, among 17 non-DOPs, 82.4% species belong to Group a. The analogs that exhibited the strongest potential against all of the cell lines were clustered in Group c (Figure 2). This cluster included GO-Y078, 079, 030, 097, and 098. All of these compounds had at least 10 times higher growth-suppressive potential than curcumin. For example, the value of GO-Y030 reached 76 times, GO-Y078 84 times, and GO-Y079 29 times higher than curcumin against GCIY. In the solution, as each ethoxyethyl group of GO-Y079 and 097 is predicted to be hydrolyzed in an acidic environment, and GO-Y079 and GO-Y097 can convert themselves to 078 and 098, respectively. Essentially, GO-Y078 is nearly identical to 079, and 098 to 097, just as shown in the cluster analysis. For this reason, we excluded GO-Y079 and 097 from further examination. In this analysis, it has been shown A549 and MCF7 were rather resistant to curcumin analogs among them (Figure 2).

### Improvement in solubility of curcumin analogs

**Figure 3:**
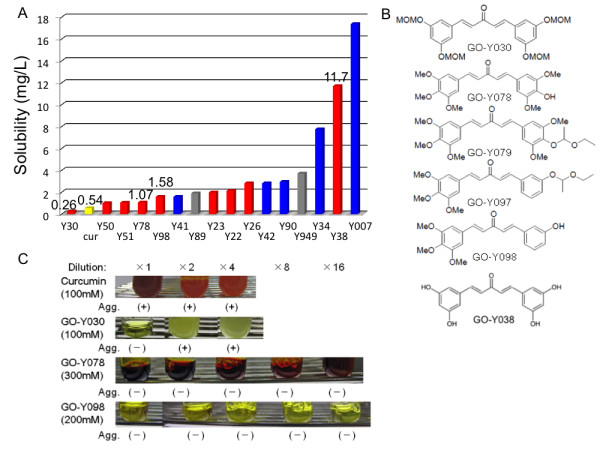
**Solubility of curcumin analogs**. (a) *in silico *prediction of solubility. The blue bar indicates analog belonging to Group a in Figure 2, and the red indicates Group b. The analogs labeled gray were not analyzed in Figure 2. (b) Chemical structure of DOPs bearing the lowest IC_50 _value and higher solubility. The methoxy group is indicated as MeO, and the methoxymethoxy group indicated as MOMO. (c) Appearance of the diluted solution of curcuminoids. Curcumin and its analogs were dissolved in ethanol-cremophor EL^® ^at nearly the maximum concentration denoted in each column, then diluted serially with PBS. The dilution ratio is depicted at the top of each column. Aggregation is labeled as Agg.

The ADME profiles of 100 curcuminoids, including virtual analogous compounds were determined *in silico*. Concerning solubility of analogs, the predicted values of top 15 are represented in the bar graph (Figure 3a). Analogs, that bear the improved solubility than curcumin and belong to Group b at the same time, are 8 compounds including GO-Y078 and Y098. The predictive values of them are 1.98 and 2.93 times higher than curcumin, respectively. The highest predictive value is 11.7 mg/L of GO-Y038, which is 21.7 times higher than curcumin. As for GO-Y030, the solubility is 0.48 times lower than curcumin. Then, we practically examined the solubility of curcumin, GO-Y030, 078, and 098 (Figure 3b). These compounds were first dissolved in ethanol-cremophor EL^® ^and then diluted in PBS. Concentration of GO-Y078 in ethanol-cremophor EL^® ^reached the highest level among them at 300 mM. While there were no aggregations seen in GO-Y078 and 098 solutions after 4 serial dilutions, the appearance of aggregation was observed in curcumin and GO-Y030 solutions immediately after the first dilution (Figure 3c). In fact, GO-Y078 and 098 showed increased solubility, as predicted.

### Growth suppression and apoptosis induction potencies of GO-Y030, 078, and 098

**Figure 4:**
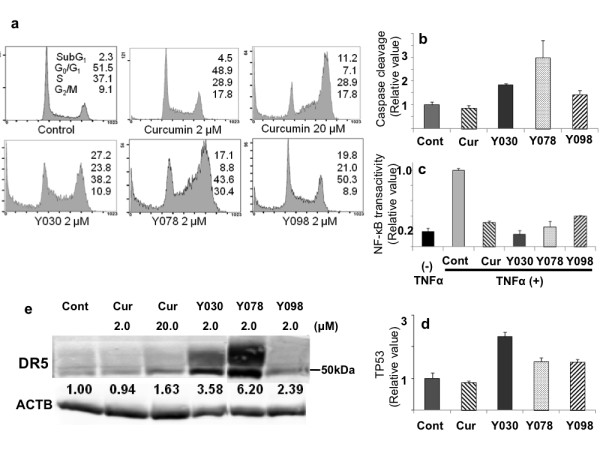
**Apoptosis induction by GO-Y030, 078, and 098**. (a) FACS analysis. Percentage of cells cycling in each phase, G_0_/G_1_, S, and G_2_/M including subG_1 _is represented in the column. (b) Effect on caspase induction. Caspase cleavage reaction was assessed by the relative expression of cleaved cytokeratin 18 against the control (= 1.0). (c) Effects on NF-κB transactivation. The relative amount of NF-κB p50 subunit binding its target DNA element, treated with 2 μM analogs in SW620 is indicated, compared to treated controls (TNFα alone, = 1.0). (d) Effects of 2 μM analogs on TP53 expression. The relative amount of TP53 is represented against the control (= 1.0). (e) Effects on DR5 expression. Relative expression value against the control (= 1.0) is represented at the bottom. β-actin is labeled as ACTB, and curcumin labeled as Cur.

To examine the growth suppression of 3 DOP analogs, GO-Y030, Y078, and Y098 clustered in Group c, the analysis of cell cycle progression in HCT116 cells was conducted by flow cytometry. Analysis of cell cycle progression indicated the increase of the subG_1 _fraction. Those treated with GO-Y030, 078, and 098 at 2 μM were 27.2%, 17.1%, and 19.8%, respectively. These values were 6.0, 3.8, and 4.4 times higher than curcumin at the same concentration (Figure 4a). GO-Y030, 078, and 098 may have a stronger potency to induce apoptosis than curcumin. To further elucidate this observation, caspase cleavage reactions were assessed. At a dose of 2 μM, the amount of cleaved product in GO-Y030, 078, and 098 treatments were 2.12 ± 0.07, 3.47 ± 0.84 and 1.67 ± 0.19 times higher, respectively, than curcumin. GO-Y030, 078, and 098 were also considered to be potent inducers of apoptosis (Figure 4b).

### GO-Y030, 078, and 098 can induce apoptosis through numerous mechanisms just as curcumin

Generally, cancer cells have some breakdown in key apoptosis induction mechanisms. These include the inactivation of a cohort of NF-κB target genes, as well as inactivation of the TP53 pathway, and extrinsic apoptotic pathways. Hyperactivated NF-κB in cancer cells leads to protection from apoptotic cell death via its targets including IAPs. It is well documented that curcumin can inhibit NF-κB transactivation [3,4]. The inhibitory effects of NF-κB transactivation in 3 DOP analogs, were examined by ELISA. In this assay, NF-κB transactivation was estimated by the amount of p50 subunit binding to its DNA consensus sequence. NF-κB p50 activates a set of target genes in response to certain inflammatory signals, such as TNFα. In this study, curcumin and DOP analogs decreased TNFα-induced NF-κB transactivation at a dose of 2 μM. The relative values of transactivation with curcumin, GO-Y030, 078, and 098 were 0.32 ± 0.02, 0.16 ± 0.05, 0.26 ± 0.07, and 0.40 ± 0.01 compared to the control (= 1.0) (Figure 4c). These DOPs were shown to have comparative potency to curcumin in the inhibition of NF-κB transactivation.

The p53 gene is altered in nearly half of human cancers, usually by point mutations, and results in the loss of ability to induce pro-apoptotic genes. Following 24 h of treatment, TP53 induction was assessed in HCT116 cells bearing wild-type p53 as a control. After treatment with a 2 μM dose, the TP53 expression in curcumin-treated cells was similar to the control level, while the levels of TP53 in GO-Y030, 078, and 098 were increased 2.32, 1.54, and 1.52 times higher, respectively, than the control. (Figure 4d). Activating certain death receptors (DR), particularly DR4 and DR5, has been shown to selectively kill cancer cells while sparing normal cells [21]. Curcumin activates DR5, but does not activate DR4 [22]. DR5 is regulated by either a p53-dependent or -independent mechanism [23]. DR5 overexpression induces ligand-independent apoptosis [24]. The expression of DR5 was estimated in DLD-1 bearing mutant TP53, by Western blotting. After 24 h of incubation, the expression of DR5 was increased to 3.58, 6.20, and 2.39 times higher than the control, in GO-030, 078, and 098 treated cells, respectively (Figure 4e). It was shown that the apoptosis induction potency, including overexpression of TP53 and DR5 was improved, but NF-κB inhibition was not.

### Therapeutic effects of DOP analogs in an experimental mouse model

**Figure 5:**
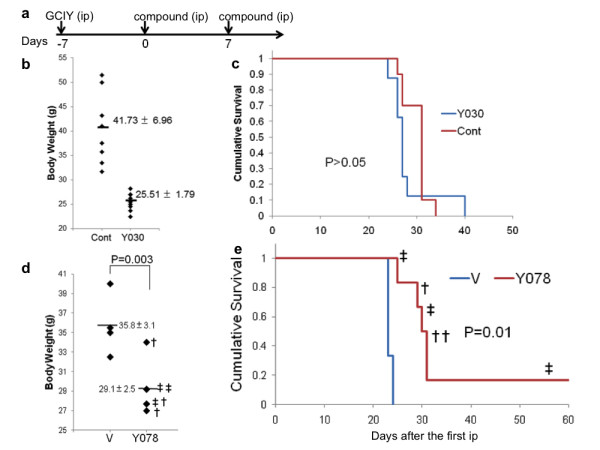
**Experimental treatment with GO-Y078 and 030 in mice**. (a) The schedule of experimental treatment. (b) Antitumor effect of GO-Y030. The body weight corresponding to the progression of PC, were evaluated. V, ethanol-cremophor, 30; Y030 155 mg/kg. (c) Survival benefit of GO-Y030. (d) Antitumor effect of GO-Y078. The body weight corresponding to the progression of PC was evaluated at the 17th day. V, ethanol-cremophor; †, 133 mg/kg; ‡, 266 mg/kg (e) Survival benefit of GO-Y078.

According to the data concerning solubility described above, GO-Y078 and 098 may have improved bioavailability. Initially, the toxicity of these compounds was examined *in vitro*. We examined the growth suppression of the primary normal liver cells, hNHeps^® ^with GO-Y030, 078, and 098. They do not affect survival nor have any toxic effects to hNHeps^® ^at doses as high as 50 μM. Then, we conducted a single IP injection, and determining the maximum tolerable dose (MTD). The MTDs of GO-Y030 and 078 were determined to be 474.5 and 400 mg/kg, respectively. Administration of these doses did not affect the animals for over 1 month. However, the MTD of GO-Y098 was rather toxic at a dose below 204 mg/kg, and this compound was excluded from further examination. The compounds were then applied to the mouse model of PC with GCIY, a gastric cancer notorious for its rapid progression [25]. The PC progresses very rapidly, and increases in ascites fluid and comparative weight gain continues until the animal dies. As a result, mortality occurs within 1 month following tumor inoculation. Experimental treatment was carried out as shown in Figure 5a. The IP administration of curcumin had no effect on the PC model, where the average body weight of untreated group reached to 164.0 ± 20.8% of that before treatment, whereas that treated with curcumin to 150.2 ± 24.0% (P = 0.18). GO-Y030 had a significant antitumor effect on PC progression (p < 0.001, Figure 5b), but no survival benefit was observed (Figure 5c). Similar to that of GO-Y030, GO-Y078 showed a significant suppression of PC progression. At the 17th day after the first treatment, the average body weight of the untreated group was 35.9 ± 2.6 g due to increased ascites fluid accumulation, while that of the treated group was significantly less at 29.1 ± 2.5 g (p = 0.003, Figure 5d). The average body weight before inoculation was 28.4 ± 0.9 g, therefore the ascites fluid accumulation was considered to be completely suppressed by GO-Y078 treatment. Survival time of the untreated group (n = 4) ranged from 21 to 23 days, with a median survival time (MST) of 22 days. Survival time of the GO-Y078 treated group (n = 6) ranged from 25 to 109 days, with a MST of 30.5 days. Treatment with GO-Y078 led to an approximate 40% increase in survival time. Log-rank analysis significantly indicated a survival benefit of GO-Y078 treatment (p = 0.01, Figure 5e). Judging from these results, the bioavailability of GO-Y078 is significantly higher than GO-Y030. In order to examine why the survival was limited in the GO-Y078 treated mice, despite the fact that the ascites fluid accumulation disappeared completely. When necropsies were performed, in nearly all of the GO-Y078-treated mice, residual cancer cells were observed to colonize around the stomach, pancreas, bile duct, and duodenum. Direct invasion to the pancreas, liver, and duodenum was thought to be the causes of death.

## Discussion

DOP analogs possess common structural feature, that is an electrophilic α,β-unsaturated ketone, which reacts with nucleophilic groups through the Michael addition. Then, the ketone binds covalently with thiol groups of cysteine residues in different proteins [26]. As thiol group does not exist in DNA, curcuminoids do not attach DNA itself, different from the cytotoxic chemotherapeutic agents. Two independent proteins can be alkylated through two α,β-unsaturated double bonds in DOP that stretch from the center ketone moiety toward both ends [27]. In this alkylation, an association of a cysteine protease, such as caspase, and its target protein may assist in the catalytic reaction of caspase, resulting in target degradation. In our previous study, we observed that DOP analogs increased caspase activity, and a caspase inhibitor inhibited the degradation of β-catenin or KRAS by DOP analogs [15]. Although this contention remains unproven, perhaps this center structure of DOP confering Michael addition is responsible for the multi-targeted nature of curcuminoids. Contribution to enhanced potency by the alteration from a diketone to a monoketone also remains to be elucidated. Shortening the chemical distance by alkylation through conversion from a di- to monoketone structure may result in an enhanced potential. Acyclic DOP analogs have an enhanced cytotoxicity [27]. Substitution in each aromatic ring could improve solubility and the resultant pharmaceutical potency. As the symmetry of the attachment group of each aromatic ring is not necessary, further improvement of solubility may be achievable by various types of substitution in this portion [17]. The hydrophilic nature and total positioning of the attached groups in a compound can reflect the potency of the analogs. In this study, the maximum IC_50 _value of DOP analogs reached a level that was 84 times lower than curcumin. The data, that curcuminoids could only affect the malignant growth, lead to the hypothesis that the growth-suppressive potential of these analogs depends on the degradation of oncoproteins that are overexpressed in malignant cells. That might be a reason why curcuminoids are rather safe to normal cells.

Furthermore, it has been shown that some of DOP analogs induce more cell death to cancer cells, compared with curcumin. Apoptosis related proteins, such as TP53 and DR5 were up-regulated with DOP analogs more efficiently than curcumin. However, the precise mechanisms of up-regulation are still unknown [28]. Cancer cells invent numerous ways to inactivate many of the apoptotic pathways. The pluripotent apoptosis induction by curcuminoids could overcome complexity of antiapoptosis in cancer cells. One of DOP analog, GO-Y078 has shown survival benefit in mouse PC model for the first time. That could not be achieved by GO-Y030. Although these two DOPs presented the same level of IC_50 _values, the predicted solubility of GO-Y078 was 1.98 mg/L, whereas that of GO-Y030 was 0.26 mg/L. In fact, GO-Y030 was less soluble compared with GO-Y078. The difference of solubility between GO-Y030 and GO-Y078 probably influences the difference in their survival benefits in the PC models. Moreover, additional times of administration of GO-Y078 may lead to further improved survival. Combined treatment with conventional cytotoxic agents, such as 5-FU and taxanes, or a DR5 ligand, for example, tumor necrosis factor-related apoptosis-inducing ligand (TRAIL), may synergistically induce better outcomes [22]. Recently, off-target toxicity has become a problem in molecular-targeted medicines which have already been approved [29,30]. Some of the potent DOP analogs may not be as safe as dietary curcumin, shown with GO-Y098, and we should be cautious against that during development.

## Conclusions

Synthesis of DOP analogs represented by GO-Y078 can overcome low bioavailability of curcumin, yielding good lead compounds for new cancer chemotherapeutic agents bearing multi-target properties.

## Methods

### Chemicals

Chemical synthesis, physical properties and molecular formulas of the newly synthesized curcumin analogs have been previously described [15,17]. See additional file 1: Physical properties of new curcumin analogs. Curcumin (Sigma-Aldrich) and its analogs were dissolved in DMSO at 10-50 mmol/L as a stock solution. Cremophor EL^® ^was purchased from Sigma-Aldrich.

### Cell lines

GCIY, HCT116, DLD1, SW680, A549, PK1, ACHN, HUH7, OVK18, MCF7, 8505c, G361, and PC3 were obtained from the Cell Resource Center for Biomedical Research (Institute of Development, Aging and Cancer, Tohoku University, Sendai, Japan). ACHN was obtained from the American Type Culture Collection (ATCC). HuCCT1, SH10TC, and A431 were obtained from Riken Cell Bank Normal human primary hepatocytes (hNHeps^®^) were purchased from Lonza. Cells were cultured in RPMI 1640 medium containing 10% fetal bovine serum.

### Growth-suppression analysis

Growth-suppressive effects of the derivative compounds were measured in different cancer cell lines for 72 h. Cell viability was assayed by quantifying the uptake and digestion of 2-(2-methoxy-4-nitrophenyl)-3-(4-nitrophenyl)-5-(2,4-disulfophenyl)-2H-tetrazolium monosodium salt according to the manufacturer's instructions (Dojindo Laboratories) by 96-well plate reader, SpectraMax M2e (Molecular Devices). The percentage cell growth of the control, which was treated with 1% DMSO alone, was calculated and plotted, and the mean growth-inhibitory concentration (IC_50_) value was then determined.

### Cluster analysis

Clustering analysis among analogs was performed with MultiExperiment Viewer software (MeV) from the institute for Genomic Research at that time [18].

### Cell Cycle analysis

Cell cycle phase was determined by fluorescence-activated cell sorting analysis. Cells from the HCT116 cell line were inoculated into 6-well plates at a concentration of 5 × 10^5 ^per well, exposed to the derivative compounds at various concentrations, cultured for 24 h, and collected and sorted using a Cytomics FC500 Flow Cytometry System (Beckman Coulter, Inc.) as previously described [19]. The percentage of each cell fraction corresponding to the subG_1_, G_0_/G_1_, S, and G_2_/M phases was calculated using WinCycle Software (Beckman).

### Expression analysis

HCT116 cells treated by the analogs were cultured for 24 h, collected and lysed with lysis buffer (500 mM Tris-HCl pH 7.5, 100 mM NaCl, 2 mM EDTA, 1 mM sodium orthovanadate, and 1% NP-40). After centrifuging samples, the supernatant fraction was transferred to a fresh tube. Samples were mixed with 5× SDS sample buffer (312.5 mM Tris-HCl pH 6.8, 50% Glycerol, 25% 2-mercaptoethanol, 12.5% SDS, and 0.1% bromophenol blue) before western blotting. Antibodies used for Western blotting were anti-beta-actin monoclonal antibody (A2066, Sigma-Aldrich), anti-DR5 monoclonal antibody (Santa Cruz Biotechnology), and Alexa Fluor 680 (Invitrogen). Membranes were scanned and semi-quantification was conducted using the Odyssey detection system (LI-COR). Expression of TP53 was measured using P53 ELISA kit (Promokine) according to the manufacturer's instructions. Samples were collected using the same method as Western blotting. Twenty micrograms of protein were incubated in wells coated with anti-human p53 antibody. Biotin-conjugated anti-human p53 antibody was then added. After incubation, streptavidin conjugated with horse radish peroxidase (HRP) was added. Tetramethyl-benzine, which reacts with HRP, was then added and the absorbance was measured at 450 nm by SpectraMax M2e.

### Caspase cleavage reaction

HCT116 cells were inoculated at a concentration of 1 × 10^4 ^per well in a 96-well plate, cultured until cells were semi-confluent, and treated with the analogs for 24 h. Serum from each sample was collected, duplicated, and assessed by ELISA using HRP-conjugated M30 monoclonal antibody (M30-Apoptsense^® ^ELISA, Peviva) according to the manufacturer's instructions. Anti-M30 monoclonal antibody recognizes a soluble cytokeratin18-Asp396 molecule that becomes exposed after cleavage by caspases (caspase3, 6, 7, and 9) during apoptosis. The absorbance was measured by SpectraMax M2e at 450 nm. These analyses were conducted in triplicate.

### NF-κB transactivation

NF-κB transactivation was measured by ELISA using a NFκB p50 Transcription Factor Assay kit (Thermo Scientific) according to the manufacturer's instructions. SW620 cells were treated with the compounds with or without Tumor Necrosis Factorα (TNFα) (10 ng/ml, Sigma) for 6 hours, washed with phosphate buffered saline (PBS), and lysed with provided M-PER^® ^lysis buffer (Thermo Scientific). Whole-cell extracts containing 20 μg of protein were added with biotinylated-NF-κB consensus DNA sequence to a streptavidin-coated 96-well plate. Sample wells were incubated with the monoclonal antibody against NF-κB p50, and then incubated with HRP-conjugated secondary antibody. Luminol/enhancer solution and stable peroxide solution were added and NF-κB transactivation was detected using an LMax Luminometer (Molecular Devices).

### *In silico *ADME analysis

Absorption, distribution, metabolism, excretion (ADME), and solubility, were analyzed *in silico *by ADMEWORKS/Predictor (Fujitsu Kyushu Systems Ltd.). This analysis is commercially available and depends on a quantitative structure-activity relationship [20]. Solubility was calculated by the linear multiple regression equation and indicated by the predicted value.

### Animal experiment

C57BL/6J mice were obtained from CLEA Japan, Inc. and KSN Slc (nu/nu) mice were obtained from Japan SLC. The injection solution was prepared by first dissolving the analogs with ethanol-cremophor EL^® ^(1:1) and then diluting the solution 1:8 with PBS. C57BL/6J mice were used for the assessment of toxicity of the C5-curcuminoids. The maximum tolerable dose (MTD) was determined by a single intraperitoneal (IP) administration. Therapeutic experiments were conducted with the IP administration of 155 mg/kg of GO-Y030 (total volume 1.0 mL). IP administration of GO-Y078 was conducted with 133 mg/kg or 266 mg/kg doses. Ethanol-cremophor EL^® ^alone diluted 1:8 with PBS was injected as a control. The PC model was established by inoculating 5 × 10^6 ^GCIY cells into the abdominal cavities 6-week-old male KSN/Slc mice, 1 week prior to treatment with the 1,5-diaryl-3-oxo-1,4-pentadiene (DOP) analogs. Pathological examination including hematoxylin-eosin staining was performed as previously described [16]. All animal experiments were performed in accordance with approved institutional animal use guidelines (Tohoku University) based on international guideline.

### Statistic analysis

Each experiment was conducted three times, unless specified otherwise. Individual values were compared by Student's *t*-test. The survival curve was drawn using the Kaplan-Meier method and compared using the log rank test.

## List of abbreviations

NF-κB: nuclear factor-κB; FAP: familial adenomatous polyposis; PC: peritoneal carcinomatosis; HRP: horse radish peroxidase; TNFα: Tumor Necrosis Factor α; ADME: Absorption, distribution, metabolism, excretion; DOP: 1,5-diaryl-3-oxo-1,4-pentadiene; DR: death receptors; MTD: maximum tolerable dose

## Competing interests

The authors declare that they have no competing interests.

## Authors' contributions

CK conducted all experiments and statistical analysis. AS and HO participated in cell culture and animal experiments. HY and YI designed and synthesized all of the analogs assayed in this study. CI participated in design of the study. HS designed this study and described the manuscript. All authors read the final version of the manuscript and approved.

## Supplementary Material

Additional file 1 **Physical properties of new curcumin analogs**. The physical properties and molecular formula of the analogs are described in the file. Click here for file
