# Supplementary material for: Synthesis of 86 species of 1,5-diaryl-3-oxo-1,4-pentadienes analogs of curcumin can yield a good lead in vivo
Source: BMC Pharmacol. 2011 May 28;11:4. doi: 10.1186/1471-2210-11-4 (PMC3115866; doi:10.1186/1471-2210-11-4)
Supplement: Additional file 1 — Physical properties of new curcumin analogs. The physical properties and molecular formula of the analogs are described in the file. [file 1471-2210-11-4-S1.DOC]

additional file 1: Physical properties of new curcumin analogs

**GO-Y007**

Red oil. IR (CHCl_3_) : 3123, 1621, 1555, 1474, 1388, 1320, 1271, 1216, 1174, 1098, 1017 cm^-1^. ^1^H-NMR (400 MHz, CDCl_3_) δ : 7.51 (2H, d, *J* = 1.7 Hz), 7.47 (2H, d, *J* = 15.6 Hz), 6.91 (2H, d, *J* = 15.6 Hz), 6.68 (2H, d, *J* = 3.4 Hz), 6.47 (2H, dd, J = 3.4, 1.7 Hz). ^13^C-NMR (100 MHz, CDCl_3_) δ : 187.8, 151.4, 144.8, 129.1, 123.1, 115.8, 112.5. MS m/z : 214 (M^+^). HRMS Calcd. for C_13_H_10_O_3_ : 214.0630. Found : 214.0624. Anal. Calcd. for C_13_H_10_O_3_ : C, 72.89. H, 4.71. Found : C, 72.49. H, 4.76.

**GO-Y008**

Yellow plate (Et_2_O) : m.p. 116-118 ℃. IR (CHCl_3_) : 3019, 1666, 1608, 1492, 1446, 1273, 1216, 1164 cm^-1^. ^1^H-NMR (400 MHz, CDCl_3_) δ : 7.79 (2H, s), 7.45 (4H, d, *J* = 7.6 Hz), 7.39 (4H, m), 7.30 (2H, t, *J* = 7.2 Hz), 2.91 (4H, t, J = 6.2 Hz), 1.76 (2H, quintet, *J* = 6.2 Hz). ^13^C-NMR (100 MHz, CDCl_3_) δ : 190.1, 136.8, 136.0, 135.8, 130.2, 128.5, 128.3, 28.5, 23.0. MS m/z : 274 (M^+^). HRMS Calcd. for C_20_H_18_O : 274.1358. Found : 274.1335. Anal. Calcd. for C_20_H_18_O : C, 87.56. H, 6.61. Found : C, 87.51. H, 6.70.

**GO-Y010**

Yellow solid (MeOH) : m.p. 181-183 ℃. IR (CHCl_3_) : 3490, 1598, 1513, 1463, 1262, 1139 cm^-1^. ^1^H-NMR (400 MHz, CDCl_3_) δ : 7.25 (2H, d, *J* = 16.4 Hz), 7.03-7.13 (6H, m), 6.86 (1H, d, *J* = 8.9 Hz), 6.85 (1H, d, *J* = 8.9 Hz), 6.77 (2H, d, 16.4 Hz), 3.93 (3H, s), 3.92 (3H, s), 3.91 (3H, s), 3.90 (3H, s). ^13^C-NMR (100 MHz, CDCl_3_) δ : 155.3, 150.1, 149.7, 149.2, 149.2, 137.4, 134.9, 129.6, 129.2, 121.3, 120.6, 120.3, 114.9, 111.2, 111.1, 109.3, 109.3, 55.9. MS m/z : 369 (M^+^). HRMS Calcd. for C_21_H_23_NO_5_ : 369.1576. Found : 369.1547. Anal. Calcd. for C_21_H_23_NO_5_ : C, 68.28. H, 6.28. N, 3.79. Found : C, 67.88. H, 6.23. N, 3.78.

**GO-Y012**

Yellow plate (MeOH) : m.p. 105-107 ℃. IR (CHCl_3_) : 3027, 1650, 1625, 1591, 1495, 1448, 1345, 1195, 982 cm^-1^. ^1^H-NMR (400 MHz, CDCl_3_) δ : 7.73 (2H, d, *J* = 15.9 Hz), 7.62 (4H, m), 7.42 (6H, m), 7.08 (2H, d, *J* = 15.9 Hz). ^13^C-NMR (100 MHz, CDCl_3_) δ : 188.9, 143.3, 134.8, 130.5, 128.9, 128.4, 125.4. MS (EI) m/z : 234 (M^+^). HRMS (EI) Calcd. for C_17_H_14_O : 234.1045. Found : 234.1024. Anal. Calcd. for C_17_H_14_O : C, 87.15. H, 6.02. Found : C, 87.11. H, 6.09.

**GO-Y013**

Yellow needle (MeOH) : m.p. 130-132 ℃. IR (CHCl_3_) : 2961, 1652, 1630, 1600, 1511, 1420, 1293, 1252, 1179, 1030 cm^-1^. ^1^H-NMR (400 MHz, CDCl_3_) δ : 7.69 (2H, d, *J* = 15.7 Hz), 7.56 (4H, d, *J* = 8.6 Hz), 6.95 (2H, d, *J* = 15.7 Hz), 6.92 (4H, d, *J* = 8.6 Hz), 3.85 (6H, s). ^13^C-NMR (100 MHz, CDCl_3_) δ : 188.7, 161.4, 142.5, 130.0, 127.5, 123.4, 114.3, 55.2. MS m/z : 294 (M^+^). HRMS Calcd. for C_19_H_18_O_3_ : 294.1256. Found : 294.1248. Anal. Calcd. for C_19_H_18_O_3_ : C, 77.53. H, 6.16. Found : C, 77.48. H, 6.25.

**GO-Y015**

Yellow box (MeOH) : m.p. 134-136 ℃. IR (CHCl_3_) : 2937, 1650, 1617, 1577, 1478, 1427, 1267, 1222, 1111, 1071 cm^-1^. ^1^H-NMR (400 MHz, CDCl_3_) δ : 8.03 (2H, d, *J* = 16.2 Hz), 7.26 (2H, dd, *J* = 8.0, 1.5 Hz), 7.15 (2H, d, *J* = 16.2 Hz), 7.09 (2H, t, *J* = 8.0 Hz), 6.96 (2H, dd, *J* = 8.0, 1.5 Hz), 3.90 (6H, s), 3.89 (6H, s). ^13^C-NMR (100 MHz ,CDCl_3_) δ : 189.7, 153.2, 148.8, 137.9, 129.1, 126.9, 124.2, 119.4, 114.1, 61.3, 55.9. MS m/z : 354 (M^+^). HRMS Calcd. for C_21_H_22_O_5_ : 354.1467. Found : 354.1464. Anal. Calcd. for C_21_H_22_O_5_ : C, 71.17. H, 6.20. Found : C, 71.27. H, 6.28.

**GO-Y016**

Yellow needle (MeOH) : m.p. 123-124 ℃. IR (CHCl_3_) : 2939, 1649, 1617, 1581, 1504, 1455, 1418, 1317, 1277, 1244, 1126 cm^-1^. ^1^H-NMR (400 MHz, DMSO-*d*_6_) δ : 7.69 (2H. d, *J* = 16.0 Hz), 7.29 (2H, d, *J* = 16.0 Hz), 7.11 (4H, s), 3.84 (12H, s), 3.71 (6H, s). ^13^C-NMR (100 MHz, DMSO-*d*_6_) δ : 188.2, 153.1, 142.9, 139.6, 130.3, 125.2, 106.1, 60.1, 56.1. MS m/z : 414 (M^+^). HRMS Calcd. for C_23_H_26_O_7_ : 414.1679. Found : 414.1668.

**GO-Y017**

Yellow box (MeOH) : m.p. 116-118 ℃. IR (CHCl_3_) : 1671, 1617, 1587, 1466, 1440, 1329, 1267, 1201, 1098 cm^-1^. ^1^H-NMR (400 MHz, CDCl_3_) δ : 8.13 (2H, d, *J* = 16.1 Hz), 7.71 (2H, m), 7.43 (2H, m), 7.25-7.35 (4H, m), 7.06 (2H, d, *J* = 16.1 Hz). ^13^C-NMR (100 MHz, CDCl_3_) δ : 188.7, 139.3, 135.4, 133.0, 131.2, 130.2, 127.7, 127.5, 127.1. MS m/z : 302 (M^+^). HRMS Calcd. for C_17_H_12_OCl_2_ : 302.0625. Found : 302.0227. Anal. Calcd. for C_17_H_12_OCl_2_ : C, 67.35. H, 3.99. Found : C, 67.22. H, 4.11.

**GO-Y018**

Yellow needle (CHCl_3_) : m.p. 210-212 ℃. IR (CHCl_3_) : 2253, 1793, 1652, 1620, 1586, 1562, 1486, 1382, 10967 cm^-1^. ^1^H-NMR (400 MHz, CDCl_3_) δ : 7.66 (2H, d, *J* = 15.9 Hz), 7.55 (4H, d, *J* = 8.4 Hz), 7.47 (4H, d, *J* = 8.4 Hz), 7.04 (2H, d, *J* = 15.9 Hz). ^13^C-NMR (100 MHz, CDCl_3_) δ : 188.3, 142.1, 133.7, 132.3, 129.7, 125.8, 124.9. MS m/z : 389 (M^+^). HRMS Calcd. for C_17_H_12_OBr_2_ : 389.9255. Found : 389.9210. Anal. Calcd. for C_17_H_12_OBr_2_ : C, 52.08. H, 3.08. Found : C, 51.89. H, 3.22.

**GO-Y019**

Yellow box (MeOH) : m.p. 124-126 ℃. IR (CHCl_3_) : 1645, 1612, 1487, 1463, 1436, 1336, 1246, 1185, 1105 cm^-1^. ^1^H-NMR (400 MHz, CDCl_3_) δ : 8.07 (2H, d, *J* = 16.2 Hz), 7.63 (2H, dd, *J* = 7.7, 1.4 Hz), 7.37 (2H, td, *J* = 8.2, 1.4 Hz), 7.18 (2H, d, *J* = 16.2 Hz), 6.99 (2H, t, *J* = 7.7 Hz), 6.94 (2H, d, *J* = 8.2 Hz), 3.92 (6H, s). ^13^C-NMR (100 MHz, CDCl_3_) δ : 190.0, 158.6, 138.2, 131.5, 128.7, 126.3, 124.0, 120.7, 111.2, 55.5. MS m/z : 294 (M^+^). HRMS Calcd. for C_19_H_18_O_3_ : 294.1256. Found : 294.1248. Anal. Calcd. for C_19_H_18_O_3_ : C, 77.53. H, 6.16. Found : C, 77.35. H, 6.11.

**GO-Y020**

Yellow solid (AcOEt/Hexane = 1 : 1) : m.p. 85-87 ℃. IR (CHCl_3_) : 2938, 1645, 1613, 1589, 1494, 1462, 1414, 1282, 1095 cm^-1^. ^1^H-NMR (400 MHz, CDCl_3_) δ : 7.93 (2H, d, *J* = 16.1 Hz), 7.37 (2H, d, *J* = 8.7 Hz), 7.09 (2H, d, *J* = 16.1 Hz), 6.73 (2H, d, *J* = 8.7 Hz), 3.95 (6H, s), 3.90 (6H, s), 3.89 (6H, s). ^13^C-NMR (100 MHz, CDCl_3_) δ : 189.5, 155.5, 153.5, 142.3, 137.7, 124.9, 123.3, 121.9, 107.6, 61.3, 60.8, 56.0. MS m/z : 414 (M^+^). HRMS Calcd. for C_23_H_26_O_7_ : 414.1679. Found : 414.1679.

**GO-Y021**

Orange box (EtOH) : m.p. 173-175 ℃. IR (CHCl_3_) : 2937, 1639, 1598, 1511, 1285, 1212, 1096, 1030 cm^-1^. ^1^H-NMR (400 MHz, CDCl_3_) δ : 8.04 (2H, d, *J* = 16.2 Hz), 7.13 (2H, s), 7.03 (2H, d, *J* = 16.2 Hz), 6,52 (2H, s), 3.95 (6H, s), 3.91 (6H, s), 3.90 (6H, s). ^13^C-NMR (100 MHz, CDCl_3_) δ : 189.6, 154.3, 152.3, 143.3, 137.5, 124.0, 115.7, 111.0, 96.9, 56.5, 56.4, 56.1. MS m/z : 414 (M^+^). HRMS Calcd. for C_23_H_26_O_7_ : 414.1679. Found : 414.1664. Anal. Calcd. for C_23_H_26_O_7_ : C, 66.65. H, 6.32. Found : C, 66.52. H, 6.27.

**GO-Y022**

Yellow solid (MeOH) : m.p. 68-70 ℃. IR (CHCl_3_) : 3389, 1639, 1588, 1510, 1428, 1268, 1183, 1105 cm^-1^. ^1^H-NMR (400 MHz, DMSO-*d*_6_) δ : 9.65 (2H, s), 7.64 (2H, d, *J* = 15.9 Hz), 7.36 (2H, s), 7.19 (2H, d, *J* = 8.2 Hz), 7.14 (2H, d, *J* = 15.9 Hz), 6.82 (2H, d, *J* = 8.2 Hz), 3.84 (6H, s). ^13^C-NMR (100 MHz, DMSO-*d*_6_) δ : 188.0, 149.4, 148.0, 142.7, 126.3, 123.3, 123.0, 115.7, 111.4, 55.7. MS m/z : 326 (M^+^). HRMS Calcd. for C_19_H_18_O_5_ : 326.1154. Found : 326.1139.

**GO-Y023**

Yellow crystals (MeOH) : m.p. 194-196 ℃. IR (CHCl_3_) : 3411, 1649, 1587, 1518, 1435, 1269, 1213, 1132 cm^-1^. ^1^H-NMR (400 MHz, CDCl_3_) δ : 7.64 (2H, d, *J* = 15.8 Hz), 7.24 (2H, s), 7.12 (2H, d, *J* = 8.3 Hz), 6.93 (2H, d, *J* = 15.8 Hz), 6.87 (2H, d, *J* = 8.3 Hz), 5.65 (2H, s), 3.95 (6H, s). ^13^C-NMR (100 MHz, DMSO-*d*_6_) δ : 187.8, 149.9, 146.5, 142.5, 127.6, 123.3, 121.6, 114.2, 111.9, 55.6. MS m/z : 326 (M^+^). HRMS Calcd. for C_19_H_18_O_5_ : 326.1154. Found : 326.1139. Anal. Calcd. for C_19_H_18_O_5_ : C, 69.93. H, 5.56. Found : C, 69.74. H, 5.60.

**GO-Y025**

Yellow solid (MeOH) : m.p. 129-130 ℃. IR (CHCl_3_) : 3403, 1593, 1512, 1260, 1137, 907 cm^-1^. ^1^H-NMR (400 MHz, CDCl3) δ : 7.60 (2H, d, *J* = 15.7 Hz), 7.14 (2H, d, *J* = 8.3 Hz), 7.08 (2H, s), 6.88 (2H, d, *J* = 8.3Hz), 6.49 (2H, d, *J* = 15.7 Hz), 5.82 (1H, s), 3.93 (6H, s), 3.92 (6H, s). ^13^C-NMR (100 MHz, CDCl_3_) δ : 183.1, 150.9, 149.1, 140.3, 128.0, 122.6, 122.0, 111.1, 110.0, 101.2, 56.0, 55.9. MS m/z : 396 (M^+^). HRMS Calcd. for C_23_H_24_O_6_ : 396.1573. Found : 396.1561.

**GO-Y026**

Yellow needle (CHCl_3_) : m.p. 79-81 ℃. IR (CHCl_3_) : 3399, 2938, 1605, 1509, 1456, 1426, 1289, 1216, 1155, 1112 cm^-1^. ^1^H-NMR (400 MHz, CDCl_3_) δ : 7.66 (2H, d, *J* = 15.8 Hz), 7.94 (2H, d, *J* = 15.8 Hz), 6.87 (4H, s), 5.82 (2H, s), 3.95 (12H, s). ^13^C-NMR (100 MHz, CDCl_3_) δ : 188.4, 147.3, 143.4, 137.4, 126.4, 123.6, 105.4, 56.4. MS m/z : 386 (M^+^). HRMS Calcd. for C_21_H_22_O_7_ : 386.1366. Found : 386.1349.

**GO-Y030**

Yellow crystals (AcOEt/Hexane = 1 : 2) : m.p. 90-92 ℃. IR (CHCl_3_) : 2904, 1674, 1654, 1626, 192, 1440, 1273, 1147, 1029 cm^-1^. ^1^H-NMR (400 MHz, CDCl_3_) δ : 7.64 (2H, d, *J* = 15.9 Hz), 7.03 (2H, d, *J* = 15.9 Hz), 6.96 (4H, d, *J* = 2.2 Hz), 6.79 (2H, t, *J* = 2.2 Hz), 5.20, (8H, s), 3.50 (12H, s). ^13^C-NMR (100 MHz, CDCl_3_) δ : 188.8, 158.7, 143.1, 136.9, 126.0, 109.6, 107.2, 94.6, 56.1. MS m/z : 474 (M^+^). HRMS Calcd. for C_25_H_30_O_9_ : 474.1890. Found : 474.1887. Anal. Calcd. for C_25_H_30_O_9_ : C, 63.28. H, 6.37. Found : C, 63.23. H, 6.41.

**GO-Y031**

Yellow solid (AcOEt) : m.p. 146-148 ℃. IR (CHCl_3_) : 2916, 1649, 1618, 1583, 1502, 1455, 1420, 1319, 1277, 1246, 1185, 1154, 1127 cm^-1^. ^1^H-NMR (400 MHz, CDCl_3_) δ : 7.63 (2H, d, *J* = 16.0 Hz), 6.97 (2H, d, *J* = 16.0 Hz), 6.83 (4H, s), 5.17 (4H, s), 3.88 (12H, s), 3.60 (6H, s). ^13^C-NMR (100 MHz, CDCl_3_) δ : 188.2, 153.3, 143.0, 136.6, 130.5, 124.6, 105.3, 98.0, 57.0, 55.9. MS m/z : 474 (M^+^). HRMS Calcd. for C_25_H_30_O_9_ : 474.1890. Found : 474.1889. Anal. Calcd. for C_25_H_30_O_9_ : C, 63.28. H, 6.37. Found : C, 63.12. H, 6.36.

**GO-Y032**

Yellow plate (MeOH) : m.p. 148-150 ℃. IR (CHCl_3_) : 3019, 1598, 1251, 1140, 1215 cm^-1^. ^1^H-NMR (400 MHz, CDCl_3_) δ : 7.75 (2H, s), 7.10 (2H, dd, *J* = 8.5, 1.7 Hz), 7.02 (2H, d, *J* = 1.7 Hz), 6.90 (2H, d, *J* = 8.5 Hz), 3.92 (6H, s), 3.91 (6H, s), 2.95 (4H, t, *J* = 5.3 Hz), 1.83 (2H, quintet, *J* = 5.6 Hz). ^13^C-NMR (100 MHz, CDCl_3_) δ : 190.1, 149.7, 148.7, 136.9, 134.6, 129.1, 124.0, 113.8, 111.0, 56.0, 28.6, 23.1. MS m/z : 394 (M^+^). HRMS Calcd. for C_24_H_26_O_5_ : 394.1780. Found : 394.1777. Anal. Calcd. for C_24_H_26_O_5_ : C, 73.08. H, 6.64. Found : C, 73.01. H, 6.57.

**GO-Y033**

Yellow solid (MeOH) : m.p. 181-183 ℃. IR (CHCl_3_) : 2253, 1649, 1618, 1593, 1496, 1262, 1181, 1098 cm^-1^. ^1^H-NMR (400 MHz, CDCl_3_) δ : 7.85 (2H, d, *J* = 2.1 Hz), 7.62 (2H, d, *J* = 15.7 Hz), 7.51 (2H, dd, *J* = 8.7, 2.1 Hz), 6.93 (2H, d, *J* = 15.7 Hz), 6.92 (2H, d, *J* = 8.7 Hz), 3.95 (6H, s). ^13^C-NMR (100 MHz, CDCl_3_) δ : 188.0, 157.5, 141.3, 132.6, 129.5, 128.8, 124.3, 112.3, 111.8, 56.4. MS m/z : 450 (M^+^). HRMS Calcd. for C_19_H_16_Br_2_O_3_ : 449.9466. Found : 449.9441. Anal. Calcd. for C_19_H_16_Br_2_O_3_ : C, 50.47. H, 3.57. Found : C, 50.46. H, 3.54.

**GO-Y034**

Yellow solid (MeOH) : m.p. 110-112 ℃. IR (CHCl_3_) : 2936, 1651, 1595, 1510, 1420, 1261, 1158, 1024 cm^-1^. ^1^H-NMR (400 MHz, CDCl_3_) δ : 7.77 (1H, d, *J* = 15.6 Hz), 7.68 (1H, dd, *J* = 8.4, 2.0 Hz), 7.63 (1H, d, *J* = 1.9 Hz), 7.42 (1H, d, *J* = 15.6 Hz), 7.25 (1H, dd, *J* = 8.4, 2.0 Hz), 7.17 (1H, d, *J* = 1.9 Hz), 6.94 (1H, d, *J* = 8.4 Hz), 6.91 (1H, d, *J* = 8.4 Hz), 3.98 (3H, s), 3.97 (3H, s), 3.96 (3H, s), 3.93 (3H, s). ^13^C-NMR (100 MHz, CDCl_3_) δ : 187.8, 152.4, 150.6, 148.6, 143.5, 131.0, 127.5, 122.3, 119.2, 110.7, 110.4, 109.8, 109.5, 55.8, 55.7. MS m/z : 328 (M^+^). HRMS Calcd. for C_19_H_20_O_5_ : 328.1311. Found : 328.1298. Anal. Calcd. for C_19_H_20_O_5_ : C, 69.50. H, 6.14. Found : C, 69.46. H, 6.20.

**GO-Y035**

Yellow solid. IR (CHCl_3_) : 3029, 2399, 1531, 1423, 1216 cm^-1^. ^1^H-NMR (400 MHz, CDCl_3_) δ : 8.51 (2H, s), 8.27 (2H, d, *J* = 7.6 Hz), 7.91 (2H, d, *J* = 7.6 Hz), 7.80 (2H, d, *J* = 16.1 Hz), 7.63 (2H, t, *J* = 7.6 Hz), 7.20 (2H, d, *J* = 16.1 Hz). MS m/z : 324 (M^+^). HRMS Calcd. for C_17_H_12_N_2_O_5_ : 324.0746. Found : 324.0729.

**GO-Y036**

Yellow solid (MeOH) : m.p. 153-155 ℃. IR (CHCl_3_) : 2953, 1659, 1596, 1513, 1247, 1139, 1024 cm^-1^. ^1^H-NMR (400 MHz, CDCl_3_) δ : 7.75 (2H, s), 7.10 (2H, dd, *J* = 8.3, 1.9 Hz), 7.00 (2H, d, *J* = 1.9 Hz), 6.91 (2H, d, *J* = 8.3 Hz), 3.92 (6H, s), 3.91 (6H, s), 3.08 (2H, d, *J* = 12.2 Hz), 2.51 (2H, dd, *J* = 12.2, 11.9 Hz), 1.90 (1H, m), 1.10 (3H, d, *J* = 6.3 Hz). ^13^C-NMR (100 MHz, CDCl_3_) δ : 184.7, 149.5, 148.5, 136.9, 133.6, 128.9, 123.7, 113.8, 110.8, 55.9, 36.6, 29.4, 21.8. MS m/z : 408 (M^+^). HRMS Calcd. for C_25_H_28_O_5_ : 408.1937. Found : 408.1938. Anal. Calcd. for C_25_H_28_O_5_ : C, 73.51. H, 6.91. Found : C, 73.11. H, 7.07.

**GO-Y037**

Yellow oil. IR (CHCl_3_) : 2931, 1594, 1511, 1251, 1135 cm^-1^. ^1^H-NMR (400 MHz, CDCl_3_) δ : 7.67 (2H, s), 7.06 (2H, dd, *J* = 8.5, 1.8 Hz), 6.98 (2H, d, *J* = 1.8 Hz), 6.90 (2H, d, *J* = 8.5 Hz), 4.79 (2H, s), 3.92 (6H, s), 3.90 (6H, s), 3.64 (2H, s), 2.62 (4H, m), 2.06 (1H, d, *J* = 13.2 Hz), 1.97 (1H, dt, *J* = 13.2, 3.0 Hz). ^13^C-NMR (100 MHz, CDCl_3_) δ : 190.2, 194.4, 148.6, 142.0, 139.1, 135.8, 128.6, 123.3, 113.7, 113.3, 110.9, 56.0, 55.9, 55.8, 55.8, 41.2, 33.8, 33.2. MS m/z : 446 (M^+^). HRMS Calcd. for C_28_H_30_O_5_ : 446.2093. Found : 446.2088.

**GO-Y038**

Yellow crystals (MeOH) : m.p. 298-300 ℃. IR (CHCl_3_) : 3289, 1599, 1515, 1215 cm^-1^. ^1^H-NMR (400 MHz, CD_3_OD) δ : 7.58 (2H, d, *J* = 15.9 Hz), 7.07 (2H, d, *J* = 15.9 Hz), 6.61 (4H, d, *J* = 1.7 Hz), 6.34 (2H, t, *J* = 1.7 Hz). ^13^C-NMR (100 MHz, CD_3_OD) δ : 191.4, 159.9, 145.4, 137.8, 126.0, 107.9, 106.2. MS m/z : 298 (M^+^). HRMS Calcd. for C_17_H_14_O_5_ : 298.0841. Found : 298.0860. Anal. Calcd. for C_17_H_14_O_5_ : C, 68.45. H, 4.73. Found : C, 68.07. H, 5.01.

**GO-Y039**

Yellow box (MeOH) : m.p. 61-63 ℃. IR (CHCl_3_) : 2938, 1649, 1617, 1584, 1455, 1420, 1276, 1244, 1127 cm^-1^. ^1^H-NMR (400 MHz, CDCl_3_) δ : 7.65 (2H, d, *J* = 15.8 Hz), 6.97 (2H, d, *J* = 15.8 Hz), 6.83 (4H, s), 5,24 (4H, s), 4.00 (4H, m), 3.88 (12H, s), 3.56 (4H, m), 3.37 (6H, s). ^13^C-NMR (100 MHz, CDCl_3_) δ : 188.3, 153.4, 143.2, 136.6, 130.6, 124.7, 105.3, 96.9, 71.6, 68.5, 59.0, 56.0. MS m/z : 562 (M^+^). HRMS Calcd. for C_29_H_38_O_11_ : 562.2414. Found : 562.2414.

**GO-Y040**

Yellow crystals (MeOH) : m.p. 100-101 ℃. IR (CHCl_3_) : 2955, 1649, 1618, 1597, 1508, 1254, 1154, 1075 cm^-1^. ^1^H-NMR (400 MHz, CDCl_3_) δ : 7.66 (2H, d, *J* = 15.9 Hz), 7.45 (2H, d, *J* = 2.0 Hz), 7.24 (2H, dd, *J* = 8.5, 2.0 Hz), 7.18 (2H, d, *J* = 8.5 Hz), 6.96 (2H, d, *J* = 15.9 Hz), 5.29 (2H, s), 5.29 (2H, s), 3.56 (6H, s), 3.53 (6H, s). ^13^C-NMR (100 MHz, CDCl_3_) δ : 188.5, 149.2, 147.3, 142.6, 129.2, 124.2, 123.9, 116.0, 115.6, 95.4, 95.0, 56.4, 56.3. MS m/z : 474 (M^+^). HRMS Calcd. for C_25_H_30_O_9_ : 474.1890. Found : 474.1886. Anal. Calcd. for C_25_H_30_O_9_ : C, 63.28. H, 6.37. Found : C, 63.06. H, 6.45.

**GO-Y041**

White solid (MeOH) : m.p. 85-86 ℃. IR (CHCl_3_) : 2935, 1710, 1590, 1516, 1464, 1261, 1156, 1028 cm^-1^. ^1^H-NMR (400 MHz, CDCl_3_) δ : 6.76 (2H, d, *J* = 8.4 Hz), 6.69 (2H, s), 6.67 (2H, d, *J* = 8.4 Hz), 3.85 (6H, s), 3.85 (6H, s), 2.83 (4H, t, *J* = 7.6 Hz), 2.69 (4H, t, *J* = 7.6 Hz). ^13^C-NMR (100 MHz, CDCl_3_) δ : 209.2, 148.7, 147.2, 133.5, 120.0, 111.6, 111.1, 55.9, 55.8, 44.9, 29.4. MS m/z : 358 (M^+^). HRMS Calcd. for C_21_H_26_O_5_ : 358.1780. Found : 358.1761. Anal. Calcd. for C_21_H_26_O_5_ : C, 70.37. H, 7.31. Found : C, 70.37. H, 7.30.

**GO-Y042**

White solid (MeOH) : m.p. 87-89 ℃. IR (CHCl_3_) : 3518, 2935, 1590, 1463, 1417, 1260, 1235, 1139, 1027 cm^-1^. ^1^H-NMR (400 MHz, CDCl_3_) δ : 6.78 (2H, d, *J* = 8.6 Hz), 6.72 (2H, d, *J* = 8.6 Hz), 6.71 (2H, s), 3.86 (6H, s), 3.85 (6H, s), 3.68 (1H, m), 2.73 (2H, m), 2.61 (2H, m), 1.77 (4H, m), 1.44 (1H, s). ^13^C-NMR (100 MHz, CDCl_3_) δ : 148.7, 147.1, 134.5, 120.0, 111.6, 111.2, 70.9, 55.9, 55.8, 39.5, 31.7. MS m/z : 360 (M^+^). HRMS Calcd. for C_21_H_28_O_5_ : 360.1937. Found : 360.1938. Anal. Calcd. for C_21_H_28_O_5_ : C, 69.98. H, 7.83. Found : C, 69.94. H, 7.75.

**GO-Y044**

Yellow crystals (AcOEt/Hexane) : m.p. 138-139 ℃. IR (CHCl_3_) : 3495, 2939, 1647, 1617, 1583, 1502, 1454, 1420, 1279, 1126 cm^-1^. ^1^H-NMR (400 MHz, CDCl_3_) δ : 7.67 (2H, d, *J* = 16.0 Hz), 6.98 (2H, d, *J* = 16.0 Hz), 6.86 (4H, s), 4.19 (4H, m), 3.93 (12H, s), 3.74 (4H, m), 3.33 (2H, t, *J* = 6.6 Hz). ^13^C-NMR (100 MHz, CDCl_3_) δ : 188.3, 153.5, 143.2, 138.7, 130.7, 125.0, 105.5, 75.5, 61.4, 56.2. MS m/z : 474 (M^+^). HRMS Calcd. for C_25_H_30_O_9_ : 474.1890. Found : 474.1898. Anal. Calcd. for C_25_H_30_O_9_ : C, 63.28. H, 6.37. Found : C, 63.10. H, 6.34.

**GO-Y045**

Yellow needle (CHCl_3_) : m.p. 192-193 ℃. IR (CHCl_3_) : 3019, 1590, 1430, 1218 cm^-1^. ^1^H-NMR (400 MHz, CDCl_3_) δ : 7.56 (2H, d, *J* = 15.8 Hz), 7.17 (2H, s), 7.05 (2H, d, *J* = 8.3 Hz), 6.86 (2H, d, *J* = 8.3 Hz), 6.48 (2H, d, *J* = 15.8 Hz), 5.78 (1H, s), 5.63 (2H, s), 3.94 (6H, s). ^13^C-NMR (100 MHz, DMSO-*d*_6_) δ : 182.9, 149.9, 146.6, 140.3, 127.5, 121.5, 121.3, 114.0, 111.9, 101.0, 55.6. MS m/z : 368 (M^+^). HRMS Calcd. for C_21_H_20_O_6_ : 368.1260. Found : 368.1266.

**GO-Y046**

Yellow amorphous. IR (CHCl_3_) : 2938, 1647, 1617, 1583, 1509, 1462, 1420, 1266, 1127 cm^-1^. ^1^H-NMR (400 MHz, CDCl_3_) δ : 7.70 (1H, d, *J* = 15.7 Hz), 7.65 (1H, d, *J* = 15.7 Hz), 7.21 (1H, dd, *J* = 8.3, 1.9 Hz), 7.14 (1H, d, *J* = 1.9 Hz), 6.98 (1H, d, *J* = 15.7 Hz), 6.96 (1H, d, *J* = 15.7 Hz), 6.90 (1H, d, *J* = 8.3 Hz), 6.85 (2H, s), 3.95 (3H, s), 3.93 (3H, s), 3.92 (6H, s), 3.90 (3H, s). ^13^C-NMR (100 MHz, CDCl_3_) δ : 188.5, 153.4, 151.4, 149.3, 143.3, 142.9, 140.3, 130.3, 127.8, 124.9, 123.4, 123.1, 111.1, 110.0, 105.6, 60.9, 56.2, 56.0, 55.9. MS m/z : 384 (M^+^). HRMS Calcd. for C_22_H_24_O_6_ : 384.1573. Found : 384.1563.

**GO-Y047**

Yellow amorphous. IR (CHCl_3_) : 2935, 1647, 1599, 1510, 1258, 1172, 1139, 1097, 1024 cm^-1^. ^1^H-NMR (400 MHz, CDCl_3_) δ : 7.71 (1H, d, *J* = 15.7 Hz), 7.68 (1H, d, *J* = 15.7 Hz), 7.58 (2H, d, *J* = 8.6 Hz), 7.20 (1H, dd, *J* = 8.6, 2.0 Hz), 7.14 (1H, d, *J* = 2.0 Hz), 6.98 (1H, d, *J* = 15.7 Hz), 6.93 (1H, d, *J* = 15.7 Hz), 6.93 (2H, d, *J* = 8.6 Hz), 6.90 (1H, d, *J* = 8.6 Hz), 3.95 (3H, s), 3.93 (3H, s), 3.86 (3H, s). ^13^C-NMR (100 MHz, CDCl_3_) δ : 188.6, 161.4, 151.1, 149.1, 142.8, 142.6, 130.0, 127.8, 127.5, 123.8, 123.1, 123.0, 114.3, 111.0, 109.7, 56.0, 55.9, 55.4. MS m/z : 324 (M^+^). HRMS Calcd. for C_20_H_20_O_4_ : 324.1362. Found : 324.1342.

**GO-Y048**

Yellow plate (AcOEt) : m.p. 162-164 ℃. IR (CHCl_3_) : 3272, 1939, 1646, 1620, 1586, 1500, 1455, 1419, 1320, 1281, 1244, 1128 cm^-1^. ^1^H-NMR (400 MHz, CDCl_3_) δ : 7.66 (2H, d, *J* = 15.9 Hz), 6.98 (2H, d, *J* = 15.9 Hz), 6.85 (4H, s), 4.79 (4H, d, *J* = 2.4 Hz), 3.92 (12H, s), 2.45 (2H, t, *J* = 2.4 Hz). ^13^C-NMR (100 MHz, CDCl_3_) δ : 188.4, 153.8, 143.3, 137.8, 131.0, 125.0, 105.6, 79.0, 75.1, 60.0, 56.3. MS m/z : 462 (M^+^). HRMS Calcd. for C_27_H_26_O_7_ : 462.1679. Found : 462.1695. Anal. Calcd. for C_27_H_26_O_7_ : C, 70.12. H, 5.67. Found : C, 70.07. H, 5.47.

**GO-Y049**

Yellow amorphous. IR (CHCl_3_) : 2938, 1649, 1618, 1584, 1510, 1420, 1322, 1253, 1173, 1127, 1099 cm^-1^. ^1^H-NMR (400 MHz, CDCl_3_) δ : 7.70 (1H, d, *J* = 15.9 Hz), 7.63 (1H, d, *J* = 15.8 Hz), 7.56 (2H, d, *J* = 8.8 Hz), 6.97 (1H, d, *J* = 15.9 Hz), 6.95 (1H, d, *J* = 15.8 Hz), 6.91 (2H, d, *J* = 8.8 Hz), 6.83 (2H, s), 3.91 (6H, s), 3.89 (3H, s), 3.84 (3H, s). ^13^C-NMR (100 MHz, CDCl_3_) δ : 188.5, 161.6, 153.4, 142.9, 142.8, 140.2, 130.3, 130.0, 127.4, 125.1, 123.0, 114.3, 105.5, 60.9, 56.1, 55.3. MS m/z : 354 (M^+^). HRMS Calcd. for C_21_H_22_O_5_ : 354.1467. Found : 354.1461.

**GO-Y050**

Yellow amorphous. IR (CHCl_3_) : 3409, 1643, 1582, 1509, 1263, 1137, 1100 cm^-1^. ^1^H-NMR (400 MHz, CDCl_3_) δ : 7.68 (1H, d, *J* = 15.8 Hz), 7.65 (1H, d, *J* = 15.8 Hz), 7.25 (1H, d, *J* = 1.7 Hz), 7.19 (1H, d, *J* = 8.1 Hz), 7.14 (1H, s), 7.11 (1H, d, *J* = 8.5 Hz), 6.97 (1H, d, *J* = 15.8 Hz), 6.92 (1H, d, *J* = 15.8 Hz), 6.89 (1H, d, *J* = 8.1 Hz), 6.86 (1H, d, *J* = 8.5 Hz), 5.77 (1H, s), 3.94 (3H, s), 3.93 (3H, s), 3.93 (3H, s). ^13^C-NMR (100 MHz, CDCl_3_) δ : 188.7, 151.3, 149.3, 148.7, 145.9, 143.0, 142.8, 128.6, 127.9, 124.0, 123.9, 123.0, 122.5, 113.0, 111.1, 110.6, 109.9, 56.0, 56.0, 55.9. MS m/z : 340 (M^+^). HRMS Calcd. for C_20_H_20_O_5_ : 340.1311. Found : 340.1312.

**GO-Y051**

Yellow amorphous. IR (CHCl_3_) : 3411, 1640, 1592, 1509, 1463, 1422, 1264 cm^-1^. ^1^H-NMR (400 MHz, CDCl_3_) δ : 7.69 (1H, d, *J* = 15.7 Hz), 7.68 (1H, d, *J* = 15.7 Hz), 7.19 (1H, dd, *J* = 8.2, 1.9 Hz), 7.17 (1H, dd, *J* = 8.2, 1.9 Hz), 7.13 (1H, d, *J* = 1.9 Hz), 7.11 (1H, d, *J* = 1.9 Hz), 6.95 (1H, d, *J* = 15.7 Hz), 6.94 (1H, d, *J* = 8.2 Hz), 6.93 (1H, d, *J* = 15.7 Hz), 6.88 (1H, d, *J* = 8.2 Hz), 6.10 (1H, s), 3.94 (3H, s), 3.94 (3H, s), 3.92 (3H, s). ^13^C-NMR (100 MHz, CDCl_3_) δ : 188.7, 151.3, 149.2, 148.2, 146.8, 143.2, 143.0, 127.8, 127.4, 123.6, 123.3, 123.3, 123.0, 114.9, 111.1, 110.0, 109.8, 55.9, 55.9. MS m/z : 340 (M^+^). HRMS Calcd. for C_20_H_20_O_5_ : 340.1311. Found : 340.1308.

**GO-Y052**

Yellow oil. IR (CHCl_3_) : 2974, 1646, 1617, 1595, 1509, 1262, 1140, 1098, 1024 cm^-1^. ^1^H-NMR (400 MHz, CDCl_3_) δ : 7.69 (1H, d, *J* = 16.0 Hz), 7.67 (1H, d, *J* = 16.0 Hz), 7.40 (1H, d, *J* = 2.1 Hz), 7.27 (1H, dd, *J* = 8.2, 2.1 Hz), 7.20 (1H, dd, *J* = 8.5, 1.9 Hz), 7.15 (1H, d, *J* = 1.9 Hz), 6.95 (2H, d, *J* = 16.0 Hz), 6.91 (1H, d, *J* = 8.5 Hz), 6.89 (1H, d, *J* = 8.2 Hz), 5.42 (1H, q, *J* = 5.3 Hz), 3.95 (3H, s), 3.93 (3H, s), 3.90 (3H, s), 3.86 (1H, m), 3.61 (1H, m), 1.54 (3H, d, *J* = 5.3 Hz), 1.23 (3H, t, *J* = 7.0 Hz). ^13^C-NMR (100 MHz, CDCl_3_) δ : 188.6, 153.0, 151.3, 149.2, 146.0, 143.0, 142.7, 127.9, 127.8, 124.4, 123.7, 123.6, 123.0, 118.3, 111.9, 111.1, 109.9, 101.1, 61.9, 55.9, 55.9, 55.9, 20.2, 15.2. MS m/z : 412 (M^+^). HRMS Calcd. for C_24_H_28_O_6_ : 412.1886. Found : 412.1877.

**GO-Y053**

Yellow oil. IR (CHCl_3_) : 2974, 1647, 1617, 1595, 1464, 1420, 1260, 1139, 1098 cm^-1^. ^1^H-NMR (400 MHz, CDCl_3_) δ : 7.69 (1H, d, *J* = 15.7 Hz), 7.68 (1H, d, *J* = 15.7 Hz), 7.12-7.21 (5H, m), 6.98 (1H, d, *J* = 15.7 Hz), 6.96 (1H, d, *J* = 15.7 Hz), 6.88 (1H, d, *J* = 8.5 Hz), 5.44 (1H, q, *J* = 5.3 Hz), 3.94 (3H, s), 3.92 (3H, s), 3.91 (3H, s), 3.84 (1H, m), 3.57 (1H, m), 1.54 (3H, d, *J* = 5.1 Hz), 1.21 (3H, t, *J* = 7.0 Hz). ^13^C-NMR (100 MHz, CDCl_3_) δ : 188.5, 151.3, 150.7, 149.2, 148.2, 143.0, 142.7, 129.3, 127.7, 124.0, 123.5, 123.0, 122.3, 118.2, 111.0, 111.0, 100.8, 61.8, 55.8, 55.8, 20.1, 15.0. MS m/z : 397 ([M-CH_3_]^+^). HRMS Calcd. for C_23_H_25_O_6_ : 397.1651. Found : 397.1623.

**GO-Y054**

Yellow oil. IR (CHCl_3_) : 3244, 2934, 1757, 1702, 1645, 1597, 1509, 1263, 1137 cm^-1^. ^1^H-NMR (400 MHz, CDCl_3_) δ : 7.67 (1H, d, *J* = 15.7 Hz), 7.65 (1H, d, *J* = 15.7 Hz), 7.42 (1H, d, *J* = 8.4 Hz), 7.37 (1H, s), 7.19 (1H, d, *J* = 8.4 Hz), 7.14 (1H, s), 6.87-6.99 (4H, m), 6.27 (1H, s), 5.61 (1H, s), 4.48 (1H, t, *J* = 6.0 Hz), 4.32 (1H, t, *J* = 6.0 Hz), 3.93 (3H, s), 3.91 (3H, s), 3.86 (3H, s), 3.17 (1H, m), 2.89 (1H, dd, *J* = 12.7, 4.4 Hz), 2.73 (1H, d, *J* = 12.7 Hz), 2.62 (2H, t, *J* = 7.8 Hz), 1.74-1.83 (4H, m), 1.56 (2H, m). ^13^C-NMR (100 MHz, CDCl_3_) δ : 188.5, 171.5, 163.7, 152.9, 151.3, 149.2, 143.1, 141.7, 140.0, 128.1, 128.0, 127.8, 124.0, 123.8, 123.1, 122.0, 112.3, 111.1, 109.9, 61.9, 60.0, 56.0, 55.9, 55.9, 55.5, 40.5, 33.5, 28.2, 24.8. FAB m/z : 567 ([M+H]^+^). HRFAB Calcd. for C_30_H_35_O_7_N_2_S : 567.2165. Found : 567.2159.

**GO-Y055**

Yellow amorphous. IR (CHCl_3_) : 3390, 2960, 1644, 1617, 1593, 1511, 1257, 1159, 1132 cm^-1^. ^1^H-NMR (400 MHz, CDCl_3_) δ : 7.68 (2H, d, *J* = 15.9 Hz), 7.11-7.20 (5H, m), 6.92-6.98 (3H, m), 5.93 (1H, s), 5.28 (2H, s), 3.96 (3H, s), 3.94 (3H, s), 3.53 (3H, s). ^13^C-NMR (100 MHz, CDCl_3_) δ : 188.7, 149.8, 148.7, 148.2, 146.8, 143.3, 142.8, 129.2, 127.4, 124.1, 123.4, 123.3, 122.5, 115.9, 114.9, 110.7, 109.8, 95.2, 56.3, 56.0. MS m/z : 370 (M^+^). HRMS Calcd. for C_21_H_22_O_6_ : 370.1416. Found : 370.1402.

**GO-Y058**

Yellow oil. IR (CHCl_3_) : 2974, 1649, 1617, 1594, 1508, 1256, 1158, 1132, 1098, 1078, 1036 cm^-1^. ^1^H-NMR (400 MHz, CDCl_3_) δ : 7.69 (2H, d, *J* = 15.7 Hz), 7.11-7.18 (6H, m), 6.97 (1H, d, *J* = 15.7 Hz), 6.97 (1H, d, *J* = 15.7 Hz), 5.45 (1H, q, *J* = 5.3 Hz), 5.28 (2H, s), 3.95 (3H, s), 3.92 (3H, s), 3.84 (1H, m), 3.58 (1H, m), 3.49 (3H, s), 1.54 (3H, d, *J* = 5.3 Hz), 1.22 (3H, t, *J* = 7.3 Hz). ^13^C-NMR (100 MHz, CDCl_3_) δ : 188.7, 150.1, 149.9, 148.8, 148.4, 143.0, 142.9, 129.4, 129.3, 124.2, 124.1, 122.6, 122.4, 118.4, 116.0, 111.1, 110.8, 100.9, 95.2, 61.9, 56.4, 56.0, 56.0, 20.2, 15.1. MS m/z : 414 ([M-C_2_H_4_]^+^). HRMS Calcd. for C_23_H_26_O_7_ : 414.1679. Found : 414.1663.

**GO-Y059**

Yellow amorphous. IR (CHCl_3_) : 3237, 1757, 1702, 1648, 1618, 1589, 1509, 1463, 1419, 1258, 1189 cm^-1^. ^1^H-NMR (400 MHz, CDCl_3_) δ : 7.69 (1H, d, *J* = 15.8 Hz), 7.67 (1H, d, *J* = 15.8 Hz), 7.20 (1H, dd, *J* = 8.0, 2.1 Hz), 7.20 (1H, *J* = 8.0, 2.1 Hz), 7.17 (1H, d, *J* = 1.7 Hz), 7.14 (1H, d, *J* = 1.7 Hz), 7.07 (1H, d, *J* = 8.2 Hz), 7.01 (1H, d, *J* = 15.8 Hz), 6.94 (1H, d, *J* = 15.8 Hz), 6.88 (1H, d, *J* = 8.2 Hz), 6.20 (1H, s), 5.57 (1H, s), 4.49 (1H, m), 4.32 (1H, m), 3.94 (3H, s), 3.92 (3H, s), 3.88 (3H, s), 3.17 (1H, m), 2.89 (1H, dd, *J* = 12.8, 4.8 Hz), 2.74 (1H, d, *J* = 12.6 Hz), 2.63 (2H, t, *J* = 7.6), 1.54-1.83 (6H, m). ^13^C-NMR (100 MHz, CDCl_3_) δ : 188.5, 171.5, 163.7, 151.4, 151.3, 149.2, 143.5, 142.1, 141.5, 133.8, 127.7, 125.6, 123.5, 123.2, 121.4, 111.6, 111.1, 110.0, 61.9, 60.1, 55.9, 55.5, 40.5, 33.5, 28.2, 24.7.

**GO-Y060**

Yellow needle (AcOEt/Hexane = 1 : 1) : m.p. 118-120 ℃. IR (CHCl_3_) : 3258, 2360, 1649, 1618, 1583, 1501, 1455, 1419, 1317, 1278, 1244, 1127 cm^-1^. ^1^H-NMR (400 MHz, CDCl_3_) δ : 7.66 (2H, d, *J* = 15.7 Hz), 6.98 (1H, d, *J* = 15.7 Hz), 6.97 (1H, d, *J* = 15.7 Hz), 6.85 (4H, s), 4.79 (2H, d, *J* = 2.4 Hz), 3.92 (12H, s), 3.90 (3H, s), 2.45 (1H, t, *J* = 2.4 Hz). ^13^C-NMR (100 MHz, CDCl_3_) δ : 188.4, 153.8, 153.5, 143.4, 143.2, 140.5, 137.8, 131.0, 130.2, 125.0, 124.8, 105.7, 105.6, 79.1, 75.1, 61.0, 60.1, 56.3, 56.2. MS (EI) m/z : 438 (M^+^). HRMS (EI) Calcd. for C_25_H_26_O_7_ : 438.1679. Found : 438.1670. Anal. Calcd. for C_25_H_26_O_7_ : C, 68.48. H, 5.98. Found : C, 68.29. H, 6.04.

**GO-Y063**

Yellow plate (AcOEt/Hexane = 2 : 1) : m.p. 122-124 ℃. IR (CHCl_3_) : 3268, 2938, 1649, 1618, 1583, 1500, 1455, 1419, 1277, 1154, 1126 cm^-1^. ^1^H-NMR (400 MHz, CDCl_3_) δ : 7.66 (1H, d, *J* = 15.9 Hz), 7.66 (1H, d, *J* = 15.9 Hz), 6.98 (1H, d, *J* = 15.9 Hz), 6.97 (1H, d, *J* = 15.9 Hz), 6.85 (4H, s), 5.18 (2H, s), 4.78 (2H, d, *J* = 2.4 Hz), 3.92 (6H, s), 3.91 (6H, s), 3.61 (3H, s), 2.45 (1H, t, *J* = 2.4 Hz). ^13^C-NMR (100 MHz, CDCl_3_) δ : 188.4, 153.8, 153.6, 143.4, 143.2, 137.8, 137.0, 131.0, 130.7, 125.0, 124.9, 105.6, 98.2, 79.1, 75.1, 60.0, 57.2, 56.3, 56.2. MS (EI) m/z : 468 (M^+^). HRMS (EI) Calcd. for C_26_H_28_O_8_ : 468.1784. Found : 468.1786. Anal. Calcd. for C_26_H_28_O_8_ : C, 66.66. H, 6.02. Found : C, 66.43. H, 6.16.

**GO-Y065**

Yellow styloid (CHCl_3_/Hexane = 1 : 2) : m.p. 107-109 ℃. IR (CHCl_3_) : 2938, 2105, 1650, 1617, 1583, 1502, 1454, 1419, 1278, 1244, 1128 cm^-1^. ^1^H-NMR (400 MHz, CDCl_3_) δ : 7.67 (2H, d, *J* = 15.8 Hz), 6.98 (2H, d, *J* = 15.8 Hz), 6.85 (4H, s), 4.20 (4H, t, *J* = 5.3 Hz), 3.92 (12H, s), 3.57 (4H, t, *J* = 5.3 Hz). ^13^C-NMR (100 MHz, CDCl_3_) δ : 188.4, 153.5, 143.3, 138.8, 130.7, 124.9, 105.5, 71.7, 56.2, 51.1. MS (FAB) m/z : 524 (M^+^). HRMS (FAB) Calcd. for C_25_H_29_N_6_O_7_ : 525.2106. Found : 525.2106. Anal. Calcd. for C_25_H_28_N_6_O_7_ : C, 57.25. H, 5.38. N, 16.02. Found : C, 57.02. H, 5.68. N, 16.03.

**GO-Y066**

Yellow oil. IR (CHCl_3_) : 2959, 1655, 1593, 1513, 1463, 1420, 1262, 1139, 1024 cm^-1^. ^1^H-NMR (400 MHz, CDCl_3_) δ : 7.46 (1H, d, *J* = 16.0 Hz), 7.09 (1H, dd, *J* = 8.5, 1.9 Hz), 7.02 (1H, d, *J* = 1.9 Hz), 6.98 (1H, d, *J* = 2.2 Hz), 6.92 (1H, dd, *J* = 8.5, 2.2 Hz), 6.86 (1H, d, *J* = 8.2 Hz), 6.78 (1H, d, *J* = 8.2 Hz), 6.55 (1H, d, *J* = 16.0 Hz), 4.42 (1H, t, *J* = 7.1 Hz), 3.91 (3H, s), 3.91 (3H, s), 3.90 (3H, s), 3.85 (3H, s), 3.17 (2H, d, *J* = 7.1 Hz), 2.24-2.38 (2H, m), 1.54 (2H, m), 0.91 (3H, t, *J* = 7.5 Hz). ^13^C-NMR (100 MHz, CDCl_3_) δ : 196.9, 151.4, 149.2, 148.9, 148.1, 143.1, 127.2, 124.2, 123.1, 120.0, 111.0, 110.8, 110.7, 109.7, 55.9, 55.9, 55.8, 55.8, 47.6, 44.5, 33.5, 22.5, 13.4. MS (EI) m/z : 430 (M^+^). HRMS (EI) Calcd. for C_24_H_30_O_5_S : 430.1814. Found : 430.1824.

**GO-Y067**

Yellow crystals (AcOEt/Hexane = 1 : 1) : m.p. 130-132 ℃. IR (CHCl_3_) : 2938, 1653, 1596, 1458, 1426, 1285, 1207, 1156, 1066 cm^-1^. ^1^H-NMR (400 MHz, CDCl_3_) δ : 7.61 (2H, d, *J* = 15.9 Hz), 7.00 (2H, d, *J* = 15.9 Hz), 6.72 (4H, d, *J* = 2.2 Hz), 6.49 (2H, t, *J* = 2.2 Hz), 3.80 (12H, s). ^13^C-NMR (100 MHz, CDCl_3_) δ : 188.6, 160.9, 143.2, 136.5, 125.7, 106.2, 102.7, 55.4. MS (EI) m/z : 354 (M^+^). HRMS (EI) Calcd. for C_21_H_22_O_5_ : 354.1467. Found : 354.1474. Anal. Calcd. for C_21_H_22_O_5_ : C, 71.17. H, 6.26. Found : C, 70.87. H, 6.24.

**GO-Y068**

Red oil. IR (CHCl_3_) : 2937, 1628, 1557, 1509, 1408, 1256, 1154, 1132, 1055 cm^-1^. ^1^H-NMR (400 MHz, CDCl_3_) δ : 13.22 (1H, s), 7.82 (2H, s), 7.23 (1H, dd, *J* = 8.5, 1.7 Hz), 7.18 (1H, d, *J* = 8.5 Hz), 7.17 (1H, d, *J* = 1.7 Hz), 5.29 (2H, s), 4.01 (3H, s), 3.94 (3H, s), 3.89 (3H, s), 3.89 (3H, s), 3.87 (3H, s), 3.53 (3H, s). ^13^C-NMR δ : 193.5, 155.0, 153.4, 150.9, 149.9, 148.8, 143.9, 138.4, 137.3, 129.6, 124.8, 122.5, 116.0, 111.2, 111.1, 95.2, 62.2, 61.6, 61.3, 61.0, 56.4, 56.0. MS (EI) m/z : 434 (M^+^). HRMS (EI) Calcd. for C_22_H_26_O_9_ : 434.1577. Found : 434.1570.

**GO-Y069**

Orange crystals (AcOEt/Hexane = 1 : 1) : m.p. 96-98 ℃. IR (CHCl_3_) : 2938, 1624, 1593, 1555, 1495, 1464, 1409, 1342, 1285, 1200, 1152, 1096, 1057, 1012 cm^-1^. ^1^H-NMR (400 MHz, CDCl_3_) δ : 13.30 (1H, s), 8.09 (1H, d, *J* = 15.7 Hz), 7.91 (1H, d, *J* = 15.7 Hz), 7.42 (1H, d, *J* = 8.7 Hz), 6.72 (1H, d, *J* = 8.7 Hz), 4.09 (3H, s), 3.97 (3H, s), 3.92 (3H, s), 3.90 (3H, s), 3.89 (3H, s), 3.89 (3H, s), 3.86 (3H, s). ^13^C-NMR (100 MHz, CDCl_3_) δ : 193.9, 155.9, 154.9, 153.9, 153.2, 150.9, 142.5, 139.2, 138.4, 137.3, 125.3, 123.7, 122.3, 111.2, 107.7, 62.2, 61.6, 61.5, 61.3, 61.0, 60.9, 56.1. MS (EI) m/z : 434 (M^+^). HRMS (EI) CAlcd. for C_22_H_26_O_9_ : 434.1577. Found : 434.1564. Anal. Calcd. for C_22_H_26_O_9_ : C, 60.82. H, 6.03. Found : C, 60.71. H, 5.98.

**GO-Y072**

Yellow amorphous. [α]_D_^24^ +17.1°(*c* 1.5, CHCl_3_). IR (CHCl_3_) : 3243, 2929, 1704, 1582, 1502, 1455, 1419, 1318, 1277, 1127 cm^-1^. ^1^H-NMR (400 MHz, CDCl_3_) δ : 7.71 (1H, s), 7.66 (1H, d, *J* = 15.7 Hz), 7.65 (1H, d, *J* = 15.7 Hz), 6.98 (2H, d, *J* = 15.7 Hz), 6.86 (2H, s), 6.84 (2H, s), 5.82 (1H, s), 5.51 (1H, s), 5.25 (2H, s), 4.50 (1H, m), 4.37 (2H, t, *J* = 7.0 Hz), 4.33 (1H, m), 4.06 (6H, s), 4.04 (3H, s), 4.02 (6H, s), 3.16 (1H, m), 2.90 (1H, dd, *J* = 11.7, 4.9 Hz), 2.74 (1H, d, *J* = 11.7 Hz), 2.31 (2H, t, *J* = 7.5 Hz), 1.89 (2H, m), 1.30-1.71 (20H, m). ^13^C-NMR (100 MHz, CDCl_3_) δ : 188.4, 173.7, 163.5, 153.5, 153.4, 143.3, 143.2, 140.4, 138.6, 130.7, 130.2, 124.9, 124.7, 122.8, 105.6, 105.5, 66.6, 64.4, 64.4, 61.8, 60.9, 60.0, 56.1, 55.5, 51.4, 50.3, 40.6, 33.9, 30.2, 29.3, 29.3, 29.2, 29.1, 29.1, 29.0, 28.9, 28.7, 28.5, 28.3, 28.2, 26.6, 26.4, 25.8, 25.8, 24.7. MS (FAB) m/z : 864 ([M+H]^+^). HRMS (FAB) Calcd. for C_45_H_62_N_5_O_10_S : 864.4217. Found : 864.4178.

**GO-Y073**

Yellow oil. IR (CHCl_3_) : 3511, 2940, 1650, 1617, 1582, 1503, 1454, 1419, 1318, 1278, 1244, 1127 cm^-1^. ^1^H-NMR (400 MHz, CDCl_3_) δ : 7.66 (2H, d, *J* = 15.7 Hz), 6.99 (1H, d, *J* = 15.7 Hz), 6.98 (1H, d, *J* = 15.7 Hz), 6.86 (2H, s), 6.85 (2H, s), 4.18 (2H, t, *J* = 4.4 Hz), 3.92 (6H, s), 3.92 (6H, s), 3.90 (3H, s), 3.75 (2H, m), 3.35 (1H, t, *J* = 6.3 Hz). ^13^C-NMR (100 MHz, CDCl_3_) δ : 188.3, 153.5, 153.4, 143.4, 143.0, 140.4, 138.6, 130.7, 130.2, 125.0, 124.7, 105.6, 105.4, 75.5, 61.4, 60.9, 56.2. MS (EI) m/z : 444 (M^+^). HRMS (EI) Calcd. for C_24_H_28_O_8_ : 444.1784. Found : 444.1787.

**GO-Y077**

Yellow oil. IR (CHCl_3_) : 2958, 1664, 1593, 1454, 1279, 1214, 1146, 1082, 1033 cm^-1^. ^1^H-NMR (400 MHz, CDCl_3_) δ : 7.72 (1H, d, *J* = 16.2 Hz), 6.87 (2H, d, *J* = 2.2 Hz), 6.77 (1H, t, *J* = 2.2 Hz), 6.74 (2H, d, *J* = 2.2 Hz), 6.64 (1H, d, *J* = 16.2 Hz), 6.61 (1H, t, *J* = 2.2 Hz), 5.12-5.17 (8H, m), 4.37 (1H, t, *J* = 7.2 Hz), 3.48 (6H, s), 3.46 (6H, s), 3.16 (2H, d, *J* = 7.0 Hz), 2.29-2.43 (2H, m), 1.51-1.58 (2H, m), 0.92 (3H, t, *J* = 7.3 Hz). ^13^C-NMR (100 MHz, CDCl_3_) δ : 196.8, 158.5, 158.3, 144.8, 142.8, 136.4, 126.8, 109.5, 109.2, 107.2, 103.5, 94.5, 94.5, 56.1, 56.0, 47.3, 44.5, 33.6, 22.5, 13.4. MS (EI) m/z : 550 (M^+^). HRMS (EI) Calcd. for C_28_H_38_O_9_S : 550.2237. Found : 550.2241.

**GO-Y078**

Yellow amorphous. IR (CHCl_3_) : 3389, 2939, 1645, 1583, 1505, 1455, 1421, 1283, 1153, 1125 cm^-1^. UV (CHCl_3_) 380 nm. ^1^H-NMR (400 MHz, CDCl_3_) δ : 7.66 (1H, d, *J* = 15.7 Hz), 7.65 (1H, d, *J* = 15.9 Hz), 6.98 (1H, d, *J* = 15.7 Hz), 6.94 (1H, d, *J* = 15.9 Hz), 5.99 (1H, s), 3.93 (6H, s), 3.91 (6H, s), 3.90 (3H, s). ^13^C-NMR (100 MHz, CDCl_3_) δ : 188.4, 153.4, 147.2, 143.7, 142.9, 140.3, 137.5, 130.3, 126.2, 124.8, 123.4, 105.5, 105.4, 60.9, 56.3, 56.1. MS (EI) m/z : 400 (M^+^). HRMS (EI) Calcd. for C_22_H_24_O_7_ : 400.1522. Found : 400.1502.

**GO-Y079**

Yellow oil. IR (CHCl_3_) : 2938, 1650, 1617, 1582, 1501, 1419, 1277, 1244, 1128 cm^-1^. ^1^H-NMR (400 MHz, CDCl_3_) δ : 7.66 (2H, d, *J* = 15.7 Hz), 6.98 (2H, d, *J* = 15.7 Hz), 6.85 (4H, s), 5.33 (1H, q, *J* = 5.1 Hz), 3.94 (6H, s), 3.91 (3H, s), 3.90 (6H, s), 3.82 (1H, m), 3.64 (1H, m), 1.51 (3H, d, *J* = 5.1 Hz), 1.19 (3H, t, *J* = 7.3 Hz). ^13^C-NMR(100 MHz, CDCl_3_) δ : 188.4, 153.5, 153.4, 143.3, 143.2, 140.4, 137.5, 130.3, 130.2, 124.8, 124.7, 105.6, 105.6, 103.3, 62.9, 60.9, 56.2, 56.0, 20.9, 15.1. MS (EI) m/z : 472 (M^+^). HRMS (EI) Calcd. for C_26_H_31_O_8_ : 471.2008.

**GO-Y080**

Yellow oil. IR (CHCl_3_) : 2931, 1742, 1582, 1502, 1418, 1277, 1229, 1126 cm^-1^. ^1^H-NMR (400 MHz, CDCl_3_) : 7.69 (1H, s), 7.66 (1H, d, *J* = 16.1 Hz), 7.65 (1H, d, *J* = 16.1 Hz), 6.98 (2H, d, *J* = 16.1 Hz), 6.86 (2H, s), 6.84 (2H, s), 5.24 (2H, s), 4.35 (2H, t, *J* = 7.1 Hz), 4.13 (2H, t, *J* = 6.8 Hz), 3.92 (6H, s), 3.90 (3H, s), 3.89 (6H, s), 3.83 (2H, s), 3.26 (2H, s), 2.41 (3H, s), 1.89 (3H, s), 1.83 (3H, s), 1.62-1.64 (4H, m), 1.27-1.31 (12H, m). ^13^C-NMR (100 MHz, CDCl_3_) δ : 188.5, 169.6, 160.6, 160.0, 153.7, 153.5, 146.1, 144.8, 144.4, 143.4, 143.3, 140.5, 138.7, 130.8, 130.3, 125.0, 124.8, 122.8, 115.1, 112.8, 105.7, 105.6, 66.7, 66.1, 61.0, 56.3, 56.2, 56.2, 50.3, 32.7, 30.3, 29.3, 29.3, 29.1, 28.9, 28.5, 26.4, 25.8, 25.3, 11.7, 7.1, 6.9. MS (FAB) m/z : 902 ([M+H]^+^). HRMS (FAB) Calcd. for C_47_H_60_N_5_O_11_S : 902.4010. Found : 902.4011.

**GO-Y081**

Yellow amorphous. IR (CHCl_3_) : 2941, 1759, 1649, 1618, 1583, 1503, 1419, 1278, 1127 cm^-1^. ^1^H-NMR (400 MHz, CDCl_3_) δ : 7.66 (1H, d, *J* = 15.7 Hz), 7.64 (1H, d, *J* = 15.9 Hz), 6.97 (1H, d, *J* = 15.7 Hz), 6.97 (1H, d, *J* = 15.9 Hz), 6.85 (2H, s), 6.84 (2H, s), 4.70 (2H, s), 3.92 (6H, s), 3.90 (9H, s), 3.81 (3H, s). ^13^C-NMR (100 MHz, CDCl_3_) δ : 188.1, 169.3, 153.1, 152.5, 143.0, 142.7, 140.1, 138.0, 130.3, 129.9, 124.7, 124.5, 105.3, 69.1, 60.6, 55.9, 55.8, 51.7. MS (EI) m/z : 472 (M^+^). HRMS (EI) Calcd. for C_25_H_28_O_9_ : 472.1713.

**GO-Y082**

Yellow powder (CHCl_3_/Et_2_O) : m.p. 208-210 ℃. IR (CHCl_3_) : 3584, 1767, 1617, 1583, 1503, 1419, 1280, 1126 cm^-1^. ^1^H-NMR (400 MHz, CDCl_3_) δ : 7.68 (1H, d, *J* = 16.0 Hz), 7.65 (1H, d, *J* = 15.9 Hz), 7.00 (1H, d, *J* = 16.0 Hz), 6.97 (1H, d, *J* = 15.9 Hz), 6.87 (2H, s), 6.85 (2H, s), 4.65 (2H, s), 3.97 (6H, s), 3.92 (6H, s), 3.91 (3H, s). ^13^C-NMR (100 MHz, CDCl_3_) δ : 188.2, 170.1, 153.5, 152.1, 143.8, 142.5, 140.6, 138.0, 131.9, 130.0, 125.7, 124.6, 105.7, 105.3, 71.1, 61.0, 56.3, 56.2. MS (EI) m/z : 458 (M^+^). HRMS (EI) Calcd. for C_24_H_26_O_9_ : 458.1577. Found : 458.1540.

**GO-Y084**

Pale yellow oil. IR (CHCl_3_) : 1590, 1502, 1460, 1401, 1237, 1125 cm^-1^. ^1^H-NMR (400 MHz, CDCl_3_) δ : 7.14 (1H, d, *J* = 7.7 Hz), 7.06 (1H, dd, *J* = 7.7, 1.7 Hz), 7.01 (1H, d, *J* = 1.7 Hz), 6.73 (2H, s), 6.50 (2H, s), 5.68 (1H, s), 3.97 (2H, s), 3.88 (3H, s), 3.87 (6H, s), 3.83 (3H, s), 3.80 (6H, s). ^13^C-NMR (100 MHz, CDCl_3_) δ : 154.2, 153.3, 153.3, 141.0, 137.5, 136.7, 136.4, 135.7, 130.9, 126.1, 119.2, 114.3, 105.8, 104.2, 60.9, 60.8, 56.1, 56.0, 36.3. MS (EI) m/z : 440 (M^+^). HRMS (EI) Calcd. for C_25_H_28_O_7_ : 440.1835. Found : 440.1845.

**GO-Y085**

Yellow oil. IR (CHCl_3_) : 2938, 2105, 1649, 1617, 1582, 1503, 1454, 1418, 1277, 1127 cm^-1^. ^1^H-NMR (400 MHz, CDCl_3_) δ : 7.66 (2H, d, *J* = 15.9 Hz), 6.98 (1H, d, *J* = 15.9 Hz), 6.97 (1H, d, *J* = 15.9 Hz), 6.85 (4H, s), 4.19 (2H, t, *J* = 5.2 Hz), 3.92 (12H, s), 3.90 (3H, s), 3.56 (2H, t, *J* = 5.2 Hz). ^13^C-NMR (100 MHz, CDCl_3_) δ : 188.4, 153.5, 153.4, 143.4, 143.2, 140.4, 138.7, 130.7, 130.2, 124.9, 124.7, 105.6, 105.4, 71.6, 61.0, 56.2, 51.1. MS (FAB) m/z : 470 ([M+H]^+^). HRMS (FAB) Calcd. for C_24_H_28_O_7_N_3_ : 470.1927. Found : 470.1940.

**GO-Y086**

Yellow amorphous. [α]_D_^23^ + 16.4°(*c* 0.25, CHCl_3_). IR (CHCl_3_) : 3296, 2929, 1701, 1648, 1618, 1583, 1503, 1462, 1419, 1278, 1244, 1629 cm^-1^. ^1^H-NMR (500 MHz, CDCl_3_) δ : 8.04 (1H, s), 7.67 (1H, d, *J* = 15.9 Hz), 7.64 (1H, d, *J* = 15.9 Hz), 6.98 (2H, d, *J* = 15.9 Hz), 6.85 (2H, s), 6.82 (2H, s), 5.80 (1H, brs). 5.77 (1H, brs), 5.07 (1H, brs), 4.71 (2H, t, *J* = 5.0 Hz), 4.65 (2H, s), 4.50 (1H, m), 4.39 (2H, t, *J* = 5.0 Hz), 4.31 (1H, m), 3.92 (6H, s), 3.90 (3H, s), 3.85 (6H, s), 3.53 (2H, t, *J* = 6.2 Hz), 3.21 (2H, m), 3.14 (1H, m), 2.90 (1H, dd, *J* = 12.9, 5.0 Hz), 2.72 (1H, d, *J* = 12.9 Hz), 2.18 (2H, t, *J* = 7.8 Hz), 1.68-1.58 (6H, m), 1.49-1.40 (4H, m), 1.25 (12H, m). ^13^C-NMR (125 MHz, CDCl_3_) δ : 188.4, 172.9, 163.6, 153.5, 153.3, 145.2, 143.5, 143.0, 140.5, 138.2, 131.0, 130.2, 125.1, 124.7, 124.1, 105.7, 105.3, 71.3, 70.8, 64.4, 61.7, 61.0, 60.1, 56.2, 56.0, 55.5, 50.5, 40.5, 39.5, 36.0, 29.6, 29.6, 29.4, 29.4, 29.4, 29.2, 28.1, 28.1, 26.9, 26.1, 25.6. MS (FAB) m/z : 907 ([M+H]^+^). HRMS (FAB) Calcd. for C_47_H_67_N_6_O_10_S : 907.4639. Found : 907.4626.

**GO-Y087**

Pale yellow solid. IR (CHCl_3_) : 2935, 1682, 1653, 1594, 1513, 1234, 1139, 1024 cm^-1^. UV (CHCl_3_) 337 nm. ^1^H-NMR (400 MHz, CDCl_3_) δ : 7.49 (1H, d, *J* = 16.1 Hz), 7.10 (1H, d, *J* = 8.3 Hz), 7.04 (1H, s), 6.86 (1H, d, *J* = 8.3 Hz), 6.80-6.75 (3H, m), 6.61 (1H, d, *J* = 16.1 Hz), 3.90 (6H, s), 3.87 (3H, s), 3.87 (3H, s), 3.84 (3H, s), 2.96 (4H, s). ^13^C-NMR (100 MHz, CDCl_3_) δ : 199.0, 151.1, 149.0, 148.7, 147.1, 142.5, 133.7, 127.2, 124.1, 122.8, 120.0, 111.7, 111.2, 110.9, 109.5, 55.9, 55.8, 55.8, 55.7, 42.4, 29.9. MS (EI) m/z : 418 (M^+^). HRMS (EI) Calcd. for C_23_H_30_O_7_ : 418.1992. Found : 418.2004.

**GO-Y090**

Colorless oil. IR (CHCl_3_) : 3499, 2935, 1589, 1508, 1458, 1420, 1330, 1238, 1126, 1007 cm^-1^. ^1^H-NMR (400 MHz, CDCl_3_) δ : 6.42 (4H, s), 3.85 (12H, s), 3.82 (6H, s), 3.71 (1H, m), 2.76 (2H, ddd, *J* = 14.7, 9.2, 6.3 Hz), 2.63 (2H, ddd, *J* = 14.7, 9.4, 6.7 Hz), 1.84-1.77 (4H, m). ^13^C-NMR (100 MHz, CDCl_3_) δ : 153.2, 137.8, 136.2, 105.3, 71.0, 60.8, 56.1, 39.4, 32.5. MS (EI) m/z : 420 (M^+^). HRMS (EI) Calcd. for C_23_H_32_O_7_ : 420.2148. Found : 420.2150.

**GO-Y091**

Yellow oil. IR (CHCl_3_) : 2930, 1671, 1651, 1620, 1596, 1487, 1290, 1255, 1185, 1102, 1047 cm^-1^. ^1^H-NMR (400 MHz, CDCl_3_) δ : 7.70 (2H, d, *J* = 15.9 Hz), 7.33 (2H, t, *J* = 8.0 Hz), 7.21 (2H, d, *J* = 8.0 Hz), 7.13 (2H, t, *J* = 2.5 Hz), 7.06 (2H, d, *J* = 15.9 Hz), 6.96 (2H, dd, *J* = 8.0, 2.5 Hz), 3.86 (6H, s). ^13^C-NMR (100 MHz, CDCl_3_) δ : 188.7, 159.8, 143.1, 136.1, 129.8, 125.5, 121.0, 116.2, 113.2, 55.2. MS (EI) m/z : 294 (M^+^). HRMS (EI) Calcd. for C_19_H_18_O_3_ : 294.1256. Found : 294.1241.

**GO-Y092**

Yellow oil. IR (CHCl_3_) : 2939, 1646, 1615, 1582, 1496, 1463, 1416, 1269, 1127, 1097 cm^-1^. ^1^H-NMR (400 MHz, CDCl_3_) δ : 7.95 (1H, d, *J* = 16.1 Hz), 7.64 (1H, d, *J* = 15.9 Hz), 7.37 (1H, d, *J* = 8.7 Hz), 7.09 (1H, d, *J* = 16.1 Hz), 6.98 (1H, d, *J* = 15.9 Hz), 6.85 (2H, s), 6.72 (1H, d, *J* = 8.7 Hz), 3.96 (3H, s), 3.92 (6H, s), 3.92 (3H, s), 3.90 (3H, s), 3.90 (3H, s). ^13^C-NMR (100 MHz, CDCl_3_) δ : 189.0, 155.8, 153.7, 142.8, 142.4, 140.3, 138.4, 130.5, 125.2, 124.7, 123.5, 121.9, 107.7, 105.6, 61.5, 61.0, 60.9, 56.2, 56.1. MS (EI) m/z : 414 (M^+^). HRMS (EI) Calcd. for C_23_H_26_O_7_ : 414.1679. Found : 414.1687.

**GO-Y093**

Yellow box (AcOEt/Hexane = 1 : 2) : m.p. 160-162 ℃. IR (CHCl_3_) : 2928, 1681, 1593, 1462, 1250, 1212, 1101 cm^-1^. ^1^H-NMR (400 MHz, CDCl_3_) δ : 8.07 (1H, d, *J* = 16.0 Hz), 7.64 (1H, d, *J* = 16.0 Hz), 7.12 (1H, s), 7.01 (1H, d, *J* = 16.0 Hz), 6.99 (1H, d, *J* = 16.0 Hz), 6.84 (2H, s), 6.52 (1H, s), 3.94 (3H, s), 3.92 (6H, s), 3.91 (3H, s), 3.90 (6H, s). ^13^C-NMR (100 MHz, CDCl_3_) δ : 188.9, 154.4, 153.4, 152.6, 143.3, 142.4, 140.2, 138.2, 130.5, 125.0, 123.7, 115.3, 110.9, 105.5, 98.8, 60.9, 56.5, 56.3, 56.2, 56.0. MS (EI) m/z : 414 (M^+^). HRMS (EI) Calcd. for C_23_H_26_O_7_ : 414.1679. Found : 414.1663. Anal. Calcd. for C_23_H_26_O_7_ : C, 66.65. H, 6.32. Found : C, 66.36. H, 6.36.

**GO-Y094**

Yellow box (AcOEt/Hexane = 1 : 1) : m.p. 171-173 ℃. IR (CHCl_3_) : 2938, 1637, 1592, 1565, 1315, 1204, 1105 cm^-1^. ^1^H-NMR (400 MHz, CDCl_3_) δ : 8.02 (2H, d, *J* = 16.4 Hz), 7.50 (2H, d, *J* = 16.4 Hz), 6.30 (2H, s), 3.93 (6H, s), 3.92 (6H, s), 3.91 (6H, s), 3.83 (6H, s). ^13^C-NMR (100 MHz, CDCl_3_) δ : 192.1, 156.4, 155.2, 154.5, 136.5, 133.5, 127.6, 111.1, 92.1, 61.1, 61.1, 56.0, 55.9. MS (EI) m/z : 474 (M^+^). HRMS (EI) Calcd. for C_25_H_30_O_9_ : 474.1890. Found : 474.1874. Anal. Calcd. for C_25_H_30_O_9_ : C, 63.28. H, 6.37. Found : C, 63.07. H, 6.33.

**GO-Y096**

Yellow plate (MeOH) : m.p. 102-104 ℃. IR (CHCl_3_) : 2939, 1651, 1619, 1579, 1504, 1321, 1126, 1100 cm^-1^. ^1^H-NMR (400 MHz, CDCl_3_) δ : 7.85 (1H, d, *J* = 16.0 Hz), 7.66 (1H, d, *J* = 16.0 Hz), 7.62 (1H, m), 7.38 (1H, m), 7.23 (1H, d, *J* = 16.0 Hz), 7.21 (1H, m), 7.13 (1H, m), 6.97 (1H, d, *J* = 16.0 Hz), 6.85 (2H, s), 3.92, (6H, s), 3.90 (3H, s). ^13^C-NMR (100 MHz, CDCl_3_) δ : 188.8, 163.0, 160.4, 153.5, 143.7, 140.6, 135.9, 135.8, 131.9, 131.8, 130.2, 129.6, 129.6, 127.7, 127.6, 125.0, 124.6, 124.5, 123.0, 122.9, 116.4, 116.2, 105.7, 61.0, 56.3. MS (EI) m/z : 342 (M^+^). HRMS (EI) Calcd. for C_20_H_19_FO_4_ : 342.1267. Found : 342.1262. Anal. Calcd. for C_20_H_19_FO_4_ : C, 70.16. H, 5.59. Found : C, 70.08. H, 5.64.

**GO-Y097**

Yellow oil. IR (CHCl_3_) : 2976, 1650, 1619, 1581, 1504, 1320, 1127, 1102 cm^-1^. ^1^H-NMR (400 MHz, CDCl_3_) δ : 7.69 (1H, d, *J* = 15.8 Hz), 7.65 (1H, d, *J* = 15.8 Hz), 7.32 (1H, m), 7.26 (1H, m), 7.25 (1H, m), 7.08 (1H, d, *J* = 15.8 Hz), 7.06 (1H, m), 6.96 (1H, d, *J* = 15.8 Hz), 6.85 (2H, s), 5.44 (1H, q, *J* = 5.3 Hz), 3.92, (6H, s), 3.90 (3H, s), 3.79 (1H, m), 3.57 (1H, m), 1.54 (3H, d, *J* = 5.3 Hz), 1.23 (3H, t, *J* = 6.9 Hz). ^13^C-NMR (100 MHz, CDCl_3_) δ : 188.5, 157.2, 153.4, 143.4, 142.9, 140.3, 136.1, 130.1, 130.0, 125.3, 124.9, 122.0, 119.4, 116.7, 105.5, 99.4, 61.2, 61.0, 56.2, 20.2, 15.3. MS (EI) m/z : 412 (M^+^). HRMS (EI) Calcd. for C_24_H_28_O_6_ : 412.1886. Found : 412.1901.

**GO-Y098**

Yellow plate (AcOEt/Hexane = 1 : 1) : m.p. 137-139 ℃. IR (CHCl_3_) : 3353, 1644, 1617, 1582, 1504, 1274, 1126 cm^-1^. ^1^H-NMR (400 MHz, CDCl_3_) δ : 7.71 (1H, d, *J* = 16.0 Hz), 7.67 (1H, d, *J* = 16.0 Hz), 7.29 (1H, t, *J* = 7.8 Hz), 7.20 (1H, d, *J* = 7.8 Hz), 7.16 (1H, s), 7.09 (1H, d, *J* = 16.0 Hz), 6.96 (1H, d, *J* = 16.0 Hz), 6.91 (1H, d, *J* = 7.8 Hz), 6.84 (2H, s), 5.79 (1H, brs), 3.92, (6H, s), 3.91 (3H, s). ^13^C-NMR (100 MHz, CDCl_3_) δ : 189.0, 156.3, 153.5, 143.9, 143.3, 140.5, 136.3, 130.2, 130.2, 125.3, 125.0, 121.0, 117.8, 115.0, 105.7, 61.0, 56.2. MS (EI) m/z : 340 (M^+^). HRMS (EI) Calcd. for C_20_H_20_O_5_ : 340.1311. Found : 340.1295. Anal. Calcd. for C_20_H_20_O_5_ : C, 70.57. H, 5.92. Found : C, 70.57. H, 6.07.

**GO-Y099**

Yellow amorphous. IR (CHCl_3_) : 3007, 1650, 1618, 1581, 1504, 1448, 1320, 1241, 1127 cm^-1^. ^1^H-NMR (400 MHz, CDCl_3_) δ : 7.59 (1H, d, *J* = 16.0 Hz), 7.48 (1H, d, *J* = 16.0 Hz), 7.46 (6H, m), 7.31-7.21 (9H, m), 7.05 (1H, d, *J* = 8.0 Hz), 7.00 (1H, t, *J* = 8.0 Hz), 6.96 (1H, d, *J* = 1.2 Hz), 6.88 (1H, d, *J* = 16.0 Hz), 6.83 (1H, d, *J* = 16.0 Hz), 6.83 (2H, s), 6.71 (1H, dd, *J* = 8.0, 1.2 Hz), 3.92, (6H, s), 3.90 (3H, s). ^13^C-NMR (100 MHz, CDCl_3_) δ : 188.5, 156.6, 153.4, 143.7, 143.1, 142.9, 140.3, 135.1, 130.1, 128.8, 128.6, 127.7, 127.2, 125.1, 124.8, 123.0, 121.5, 120.6, 105.5, 90.7, 60.8, 56.0. MS (EI) m/z : 581 [(M-H)^+^]. HRMS (EI) Calcd. for C_39_H_33_O_5_ : 581.2328. Found : 581.2332.

**GO-Y100**

Yellow needle (AcOEt/Hexane = 1 : 1) : m.p. 107-109 ℃. IR (CHCl_3_) : 2942, 1647, 1611, 1578, 1495, 1219, 1046 cm^-1^. ^1^H-NMR (400 MHz, CDCl_3_) δ : 8.03 (2H, d, *J* = 16.2 Hz), 7.16 (2H, d, *J* = 16.2 Hz), 7.16 (2H, d, *J* = 3.0 Hz), 6.93 (2H, dd, *J* = 8.9, 3.0 Hz), 6.87 (2H, d, *J* = 8.9 Hz), 3.88, (6H, s), 3.82 (6H, s). ^13^C-NMR (100 MHz, CDCl_3_) δ : 189.9, 153.6, 153.1, 138.1, 126.4, 124.6, 117.2, 113.2, 112.5, 56.1, 55.8. MS (EI) m/z : 354 (M^+^). HRMS (EI) Calcd. for C_21_H_22_O_5_ : 354.1467. Found : 354.1469. Anal. Calcd. for C_21_H_22_O_5_ : C, 71.17. H, 6.26. Found : C, 71.05. H, 6.37.

**GO-Y101**

Yellow box (AcOEt/Hexane = 2 : 1) : m.p. 153-155 ℃. IR (CHCl_3_) : 2940, 1640, 1604, 1573, 1475, 1326, 1254, 1107 cm^-1^. ^1^H-NMR (400 MHz, CDCl_3_) δ : 8.16 (2H, d, *J* = 16.4 Hz), 7.58 (2H, d, *J* = 16.4 Hz), 7.27 (2H, t, *J* = 8.4 Hz), 6.57 (2H, d, *J* = 8.4 Hz), 3.91 (12H, s). ^13^C-NMR (100 MHz, CDCl_3_) δ : 192.6, 160.2, 133.4, 130.9, 129.2, 113.2, 103.8, 55.8. MS (EI) m/z : 354 (M^+^). HRMS (EI) Calcd. for C_21_H_22_O_5_ : 354.1467. Found : 354.1458. Anal. Calcd. for C_21_H_22_O_5_ : C, 71.17. H, 6.26. Found : C, 71.20. H, 6.26.

**GO-Y102**

Yellow oil. IR (CHCl_3_) : 2907, 1745, 1651, 1620, 1582, 1504, 1321, 1209, 1182, 1127, 1101, 1052 cm^-1^. ^1^H-NMR (400 MHz, CDCl_3_) δ : 7.70 (1H, d, *J* = 16.0 Hz), 7.66 (1H, d, *J* = 16.0 Hz), 7.43 (1H, dt, *J* = 7.8, 1.8 Hz), 7.40 (1H, t, *J* = 7.8 Hz), 7.34 (1H, t, *J* = 1.8 Hz), 7.09 (1H, dt, *J* = 7.8, 1.8 Hz), 7.08 (1H, d, *J* = 16.0 Hz), 6.94 (1H, d, *J* = 16.0 Hz), 6.85 (2H, s), 3.92, (6H, s), 3.90 (3H, s), 2.11-2.08 (9H, m), 1.82-1.75 (6H, m). ^13^C-NMR (100 MHz, CDCl_3_) δ : 188.4, 175.9, 153.4, 151.5, 143.5, 142.0, 140.5, 136.3, 130.1, 129.8, 125.9, 125.9, 124.9, 123.5, 120.8, 105.6, 60.9, 56.1, 41.0, 38.7, 36.4, 27.8. MS (EI) m/z : 502 (M^+^). HRMS (EI) Calcd. for C_31_H_34_O_6_ : 502.2355. Found : 502.2344.

**GO-Y103**

Yellow needle (AcOEt/Hexane = 1 : 1) : m.p. 140-142 ℃. IR (CHCl_3_) : 1643, 1598, 1572, 1504, 1293, 1273, 1160, 1096 cm^-1^. ^1^H-NMR (400 MHz, CDCl_3_) δ : 7.98 (2H, d, *J* = 16.1 Hz), 7.55 (2H, d, *J* = 8.6 Hz), 7.07 (2H, d, *J* = 16.1 Hz), 6.52 (2H, dd, *J* = 8.6, 2.3 Hz), 6.46 (2H, d, *J* = 2.3 Hz), 3.89 (6H, s), 3.85 (6H, s). ^13^C-NMR (100 MHz, CDCl_3_) δ : 190.0, 162.8, 160.1, 137.8, 130.2, 124.3, 117.3, 105.4, 98.4, 55.5, 55.5. MS (EI) m/z : 354 (M^+^). HRMS (EI) Calcd. for C_21_H_22_O_5_ : 354.1467. Found : 354.1451. Anal. Calcd. for C_21_H_22_O_5_ : C, 71.17. H, 6.26. Found : C, 70.95. H, 6.31.

**GO-Y104**

Yellow needle (AcOEt) : m.p. 220-222 ℃. IR (CHCl_3_) : 2938, 1633, 1595, 1455, 1320, 1204, 1157, 1117 cm^-1^. ^1^H-NMR (400 MHz, CDCl_3_) δ : 8.12 (2H, d, *J* = 16.1 Hz), 7.46 (2H, d, *J* = 16.1 Hz), 6.13 (4H, s), 3.90 (12H, s), 3.85 (6H, s). ^13^C-NMR (100 MHz, CDCl_3_) δ : 192.5, 162.6, 161.4, 133.1, 126.8, 106.8, 90.6, 55.7, 55.4. MS (EI) m/z : 414 (M^+^). HRMS (EI) Calcd. for C_23_H_26_O_7_ : 414.1679. Found : 141.1686. Anal. Calcd. for C_23_H_26_O_7_ : C, 66.65. H, 6.32. Found : C, 66.35. H, 6.33.

**GO-Y105**

Yellow oil. IR (CHCl_3_) : 2923, 1651, 1619, 1581, 1504, 1267, 1244, 1128, 1102 cm^-1^. ^1^H-NMR (400 MHz, CDCl_3_) δ : 7.69 (1H, d, *J* = 15.8 Hz), 7.65 (1H, d, *J* = 15.8 Hz), 7.30 (1H, t, *J* = 7.9 Hz), 7.18 (1H, d, *J* = 7.9 Hz), 7.13 (1H, t, *J* = 2.3 Hz), 7.08 (1H, d, *J* = 15.8 Hz), 6.96 (1H, d, *J* = 15.8 Hz), 6.94 (1H, dd, *J* = 7.9, 2.3 Hz), 6.84 (2H, s), 3.98 (2H, t, *J* = 6.6 Hz), 3.91, (6H, s), 3.90 (3H, s), 1.80 (2H, m), 1.47 (2H, m), 1.35-1.26 (20H, m), 1.88 (3H, t, *J* = 6.9 Hz). ^13^C-NMR (100 MHz, CDCl_3_) δ : 188.6, 159.5, 153.5, 143.3, 143.2, 140.4, 136.1, 130.2, 129.8, 125.3, 125.0, 120.9, 116.7, 113.9, 105.6, 68.1, 60.9, 56.2, 31.9, 29.6, 29.6, 29.6, 29.6, 29.5, 29.3, 29.2, 26.0, 22.6, 14.0. MS (EI) m/z : 536 (M^+^). HRMS (EI) Calcd. for C_34_H_48_O_5_ : 536.3502. Found : 536.3494.

**GO-Y106**

Yellow needle (AcOEt/Hexane = 1 : 1) : m.p. 116-118 ℃. IR (CHCl_3_) : 2938, 1644, 1578, 1485, 1256, 1107 cm^-1^. ^1^H-NMR (400 MHz, CDCl_3_) δ : 8.05 (2H, d, *J* = 16.3 Hz), 7.64 (2H, d, *J* = 16.3 Hz), 6.90 (2H, d, *J* = 8.9 Hz), 6.62 (2H, d, *J* = 8.9 Hz), 3.87 (6H, s), 3.87 (6H, s), 3.85 (6H, s). ^13^C-NMR (100 MHz, CDCl_3_) δ : 192.2, 153.8, 150.0, 147.1, 133.8, 130.1, 118.8, 114.4, 105.9, 60.9, 56.5, 56.0. MS (EI) m/z : 414 (M^+^). HRMS (EI) Calcd. for C_23_H_26_O_7_ : 414.1679. Found : 414.1673. Anal. Calcd. for C_23_H_26_O_7_ : C, 66.65. H, 6.32. Found : C, 66.54. H, 6.37.

**GO-Y107**

Yellow oil. IR (CHCl_3_) : 2939, 1650, 1619, 1581, 1504, 1319, 1267, 1245, 1126, 1102 cm^-1^. ^1^H-NMR (400 MHz, CDCl_3_) δ : 7.71 (1H, d, *J* = 15.9 Hz), 7.66 (1H, d, *J* = 15.9 Hz), 7.33 (1H, t, *J* = 8.0 Hz), 7.22 (1H, d, *J* = 8.0 Hz), 7.15 (1H, t, *J* = 2.0 Hz), 7.08 (1H, d, *J* = 15.9 Hz), 6.96 (1H, d, *J* = 15.9 Hz), 6.96 (1H, dd, *J* = 8.0, 2.0 Hz), 6.85 (2H, s), 3.92, (6H, s), 3.90 (3H, s), 3.86 (3H, s). ^13^C-NMR (100 MHz, CDCl_3_) δ : 188.7, 160.0, 153.5, 143.5, 143.1, 140.5, 136.2, 130.3, 130.0, 125.5, 125.0, 121.1, 116.3, 113.4, 105.7, 61.0, 56.2, 55.4. MS (EI) m/z : 354 (M^+^). HRMS (EI) Calcd. for C_21_H_22_O_5_ : 354.1467. Found : 354.1454.

**GO-Y108**

Yellow oil. IR (CHCl_3_) : 2939, 1650, 1619, 1582, 1504, 1321, 1243, 1151, 1127, 1103, 1007 cm^-1^. ^1^H-NMR (400 MHz, CDCl_3_) δ : 7.70 (1H, d, *J* = 15.9 Hz), 7.66 (1H, d, *J* = 15.9 Hz), 7.33 (1H, t, *J* = 7.6 Hz), 7.31 (1H, m), 7.26 (1H, d, *J* = 7.6 Hz), 7.09 (1H, m), 7.08 (1H, d, *J* = 15.9 Hz), 6.97 (1H, d, *J* = 15.9 Hz), 6.85 (2H, s), 5.22 (2H, s), 3.92, (6H, s), 3.90 (3H, s), 3.51 (3H, s). ^13^C-NMR (100 MHz, CDCl_3_) δ : 188.7, 157.7, 153.5, 143.5, 143.0, 140.5, 136.3, 130.3, 130.0, 125.6, 125.0, 122.3, 118.6, 115.5, 105.6, 94.5, 61.0, 56.2, 56.1. MS (EI) m/z : 384 (M^+^). HRMS (EI) Calcd. for C_22_H_24_O_6_ : 384.1573. Found : 384.1555.

**GO-Y109**

Yellow amorphous. IR (CHCl_3_) : 1651, 1622, 1581, 1504, 1419, 1320, 1212, 1127 cm^-1^. ^1^H-NMR (400 MHz, CDCl_3_) δ : 7.70 (1H, d, *J* = 15.7 Hz), 7.68 (1H, d, *J* = 15.9 Hz), 7.62 (1H, d, *J* = 7.9 Hz), 7.53 (1H, m), 7.51 (1H, t, *J* = 7.9 Hz), 7.31 (1H, dd, *J* = 7.9, 2.2 Hz), 7.12 (1H, d, *J* = 15.9 Hz), 6.95 (1H, d, *J* = 15.7 Hz), 6.86 (2H, s), 3.93, (6H, s), 3.91 (3H, s). ^13^C-NMR (100 MHz, CDCl_3_) δ : 187.9, 153.4, 149.8, 144.0, 140.6, 140.2, 137.5, 130.7, 129.9, 128.2, 127.1, 123.4, 122.5, 120.3, 118.6 (1C, q, *J* = 321.2 Hz), 105.6, 60.8, 56.0. MS (EI) m/z : 472 (M^+^). HRMS (EI) Calcd. for C_21_H_19_F_3_O_7_S : 472.4326. Found : 472.0775.

**GO-Y110**

Yellow oil. IR (CHCl_3_) : 2938, 1650, 1619, 1583, 1504, 1451, 1419, 1325, 1281, 1186, 1127, 1001 cm^-1^. ^1^H-NMR (400 MHz, CDCl_3_) δ : 7.75 (1H, d, *J* = 15.9 Hz), 7.66 (1H, d, *J* = 15.9 Hz), 7.63 (2H, m), 7.42 (3H, m), 7.11 (1H, d, *J* = 15.9 Hz), 6.97 (1H, d, *J* = 15.9 Hz), 6.85 (2H, s), 3.92, (6H, s), 3.90 (3H, s). ^13^C-NMR (100 MHz, CDCl_3_) δ : 188.7, 153.5, 143.4, 143.2, 140.5, 134.8, 130.5, 130.3, 129.0, 128.4, 125.2, 125.1, 105.6, 61.0, 56.2. MS (EI) m/z : 324 (M^+^). HRMS (EI) Calcd. for C_20_H_20_O_4_ : 324.1362. Found : 324.1340.
